# Nickel–Iron Layered Double Hydroxides/Nickel Sulfide Heterostructured Electrocatalysts on Surface-Modified Ti Foam for the Oxygen Evolution Reaction

**DOI:** 10.1021/acsami.4c08215

**Published:** 2024-09-12

**Authors:** Habib Gemechu Edao, Chia-Yu Chang, Woldesenbet Bafe Dilebo, Fikiru Temesgen Angerasa, Endalkachew Asefa Moges, Yosef Nikodimos, Chemeda Barasa Guta, Keseven Lakshmanan, Jeng-Lung Chen, Meng-Che Tsai, Wei-Nien Su, Bing Joe Hwang

**Affiliations:** † Nano-Electrochemistry Laboratory, Department of Chemical Engineering, National Taiwan University of Science and Technology, Taipei 106, Taiwan; ‡ Nano-Electrochemistry Laboratory, Graduate Institute of Applied Science and Technology, National Taiwan University of Science and Technology, Taipei 10607, Taiwan; § Sustainable Electrochemical Energy Development Center, National Taiwan University of Science and Technology, Taipei 106, Taiwan; ∥ National Synchrotron Radiation Research Center, Hsin-Chu 30076, Taiwan

**Keywords:** water splitting, multi-interface, heterostructure, synergistic effect, stability

## Abstract

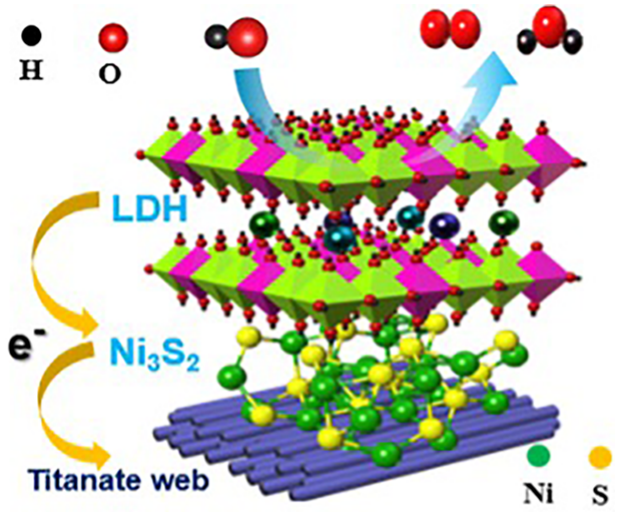

Electrochemical approaches for generating hydrogen from water splitting can be more promising if the challenges in the anodic oxygen evolution reaction (OER) can be harnessed. The interface heterostructure materials offer strong electronic coupling and appropriate charge transport at the interface regions, promoting accessible active sites to prompt kinetics and optimize the adsorption–desorption of active species. Herein, we have designed an efficient multi-interface-engineered Ni_3_Fe_1_ LDH/Ni_3_S_2_/TW heterostructure on in situ generated titanate web layers from the titanium foam. The synergistic effects of the multi-interface heterostructure caused the exposure of rich interfacial electronic coupling, fast reaction kinetics, and enhanced accessible site activity and site populations. The as-prepared electrocatalyst demonstrates outstanding OER activity, demanding a low overpotential of 220 mV at a high current density of 100 mA cm^–2^. Similarly, the designed Ni_3_Fe_1_ LDH/Ni_3_S_2_/TW electrocatalyst exhibits a low Tafel slope of 43.2 mV dec^–1^ and excellent stability for 100 h of operation, suggesting rapid kinetics and good structural stability. Also, the electrocatalyst shows a low overpotential of 260 mV at 100 mA cm^–2^ for HER activity. Moreover, the integrated electrocatalyst exhibits an incredible OER activity in simulated seawater with an overpotential of 370 mV at 100 mA cm^–2^ and stability for 100 h of operation, indicating good OER selectivity. This work might benefit further fabricating effective and stable self-sustained electrocatalysts for water splitting in large-scale applications.

## 1 Introduction

Due to the rising worldwide energy demand and the climate shift induced by the extreme use of fossil fuels, electrocatalytic water splitting, comprising the oxygen evolution reaction (OER) and hydrogen evolution reaction (HER), has received a lot of devotion in the advancement of renewable and clean H_2_ energy, owing to its CO_2_ neutrality and high energy density.^1−3^ However, OER is the bottleneck because of its slow kinetics demanding high overpotentials of OER.^4,5^ RuO_2_ and IrO_2_ are eminent benchmark catalysts for the OER.^6,7^ Nevertheless, scarcity and low stability impede their broad applications.^8^ The focus has been on alternative transition-metal-based OER electrocatalysts such as sulfides, selenides, phosphides, and layered double hydroxides (LDHs).^9^ Among them, LDHs have been considered potential OER electrocatalysts^10,11^ due to their flexible layered structure, tunable compositions, and readily exchangeable intercalated anions.^12^ Explicitly, NiFe LDH, in which Fe^3+^ is integrated into Ni(OH)_2_, is the most active non-noble OER electrocatalyst.^13^ However, it has intrinsically poor conductivity, low stability, and a limited specific surface area.^14^

In light of this, the design of a heterointerface structure and tuning of the electronic configuration have been significantly advanced as a viable approach due to their synergistic effects, interface electronic interactions, enriched active surface sites, and quick charge transfer.^15,16^ For instance, Dong et al.^17^ constructed a CeO_2_@NiFe-LDH interface heterostructure. Due to the synergistic interaction, the heterointerface catalyst revealed outstanding OER activity with an overpotential of 246 mV at a current density of 10 mA cm^–2^ and fast kinetics. The enhanced charge transfer at the heterostructured interface can adjust electronic structure, boost higher oxidation states and electrical conductivity, and significantly augment composites’ performance. Currently, Ni_3_S_2_ interface heterostructures have become an inspiring electrocatalyst for water electrolysis owing to their redox abilities and good conductivity due to the existence of networked Ni–Ni bonds.^18,19^ Wu et al.^5^ designed the Ni_3_S_2_–CeO_2_/NF hybrid heterostructure. Due to the robust interfacial electronic coupling, exposed accessible active sites, and easy charge transfer, it exhibits an OER activity with a low overpotential of 264 mV at 20 mA cm^–2^. Benefiting from the enhanced electron transfer capability near the interface sites and tuned electronic structure, interface engineering of heterostructures is an effective method for enhancing OER activity.

Besides, the catalyst–support interactions can stabilize the OER intermediate phase and lower the activation barrier.^20^ However, the majority of catalysts are synthesized in powder form. The polymer binder additives are applied to adhere the catalyst to the current collector, which covers the active sites, inhibits mass transfer, and limits electrolyte transport, affecting activity and stability.^21,22^ Moreover, numerous works have been done to integrate transition metal LDHs with conductive carbonaceous components like graphene, carbon nanotubes, and carbon paper as supporting materials.^23^ However, the impact of catalyst stability has received little attention, as carbon-based materials are frequently prone to corrosion and poor stability.^24,25^ The self-supported catalyst on conductive substrates, like nickel and copper foams, has become a common catalyst–support.^26^ Similarly, Ti-based substrates have been getting more attention because of their great mechanical assets, superior corrosion resistance, and high stability.^27,28^ For instance, Bao and his colleagues reported that the NiCo_2_O_4_@MoS_2_ heterostructure on the Ti mesh exhibits an OER overpotential of 380 mV at 100 mA cm^–2^ and high stability for 100 h.^29^ However, the overpotential is still disappointing. Further work reported that a mixed alloy of ruthenium and titanium oxide on a Ti sheet substrate for value-added anodic reaction electrolysis was stable for 36 h.^30^ Nonetheless, the stability needs further improvement, and the work does not justify how titania can be formed in situ from the Ti sheet substrate.

While there have been some inspiring achievements in interface heterostructure for OER activity, there has been no report concurrently tracking high catalytic activity and stability from the viewpoint of multi-interface-engineered heterostructures based on in situ generated titanate layers via surface modification of Ti foam (TF). Thus, it is believed that designing a highly coupled multi-interface-engineered heterostructure is a simple strategy to achieve higher intrinsic activity and augmented electronic interactions, triggering a high concentration of interfacial active regions and excellent stability. In this work contribution, the design of a robustly coupled multi-interfacial self-sustained Ni_3_Fe_1_ LDH/Ni_3_S_2_/TW heterostructure, we consider the advantage of commercially accessible Ti foam as the catalyst–support and the basis for in situ generation of titanate web layers (TW) by a simple base etching treatment that can promote the catalyst–support interactions and exceptional stability. Due to the induced synergistic effects of fast charge transfer at the rich interface sites with further enhanced electronic conductivity and intrinsic site activity, the electrocatalyst achieved great OER performance at a low overpotential of 220 mV to reach a high current density of 100 mA cm^–2^. It exhibited a low Tafel slope of 43.2 mV dec^–1^ with a long-lasting stability for 100 h. The electrode likewise displays a low overpotential of 260 mV at 100 mA cm^–2^ for the HER activity. The Ni_3_Fe_1_ LDH/Ni_3_S_2_/TW electrocatalyst was utilized as the cathode and anode for overall water splitting and achieved a 100 mA cm^–2^ current density at a cell voltage of 1.56 V and remained stable for 85 h. Notably, the electrocatalyst demonstrated remarkable activity at a low cell voltage of 1.6 V to attain 100 mA cm^–2^ in the seawater simulation. It sustains stability for 100 h of operation, revealing good OER selectivity and stability.

## 2 Experimental Section

### 2.1 Surface Modification of Ti Foam

The surface of Ti foam was treated using a previously described approach for Ti sheets with simple modifications.^4^ Precisely, TF of 1.60 mm thickness was sonicated in 3 M HCl, acetone, deionized water (DIW), and ethanol for 15 min. The cleaned TF was held vertically in a 100 mL Teflon-lined autoclave filled with 1 M NaOH in 40 mL of solution and heated at 220 °C for 16 h. The produced sodium titanate (Na_2_Ti_3_O_7_) nanoweb during hydrothermal treatment was dipped into 0.5 M HCl solution to replace Na^+^ with H^+^ ions. The hydrogen titanate (H_2_Ti_3_O_7_) web layer was subsequently generated, repeatedly washed with DI water, and then desiccated in an oven at 60 °C for 12 h.

### 2.2 Synthesis of Ni_3_S_2_

The Ni_3_S_2_/TW was synthesized by adding Ni(NO)_2_·6H_2_O and CS(NH_2_)_2_ with molar ratios of 3:2 in 20 mL of aqueous solution for each. After stirring for 10 min, the solutions were combined and agitated for 30 min. The solution was placed inside a 100 mL Teflon-lined stainless-steel autoclave with a titanate web and kept at 160 °C for 12 h. After the autoclave was cooled, the sample was cleaned in ethanol and DIW and oven-dried for 6 h at 60 °C.

### 2.3 Synthesis of Ni_3_Fe_1_ LDH/Ni_3_S_2_

With a minor modification of the previously applied method for NiMn-LDHs,^13^ a similar procedure was used to synthesize the Ni_3_Fe_1_ LDH/Ni_3_S_2_/TW electrocatalyst. Typically, 40 mL of DIW was used to dissolve Ni(NO_3_)_2_·6H_2_O and Fe(NO_3_)_3_·9H_2_O by keeping molar ratios of 3:1 Ni/Fe and stirring for 10 min. Subsequently, 10 mmol of (NH_2_)_2_CO and 4 mmol of NH_4_F were added to encourage precipitation and build an alkaline environment. After stirring it for 30 min, the homogeneous Ni_3_S_2_/TW was placed inside a 100 mL Teflon-lined stainless steel autoclave and heated to 120 °C for 12 h. The sample was washed using DIW and ethanol and vacuum-dried for 12 h at 60 °C. The Ni_3_Fe_1_ LDH/TW electrocatalyst was similarly prepared on TW layers without adding Ni_3_S_2_/TW. The mass loading of the catalyst on the TF was determined at about 4.3 mg cm^–2^ based on the mass difference before and after catalyst growth, measured using a high-precision electronic balance.

## 3 Results and Discussion

### 3.1 Materials Synthesis and Support Surface Modification

**Figure 1:**
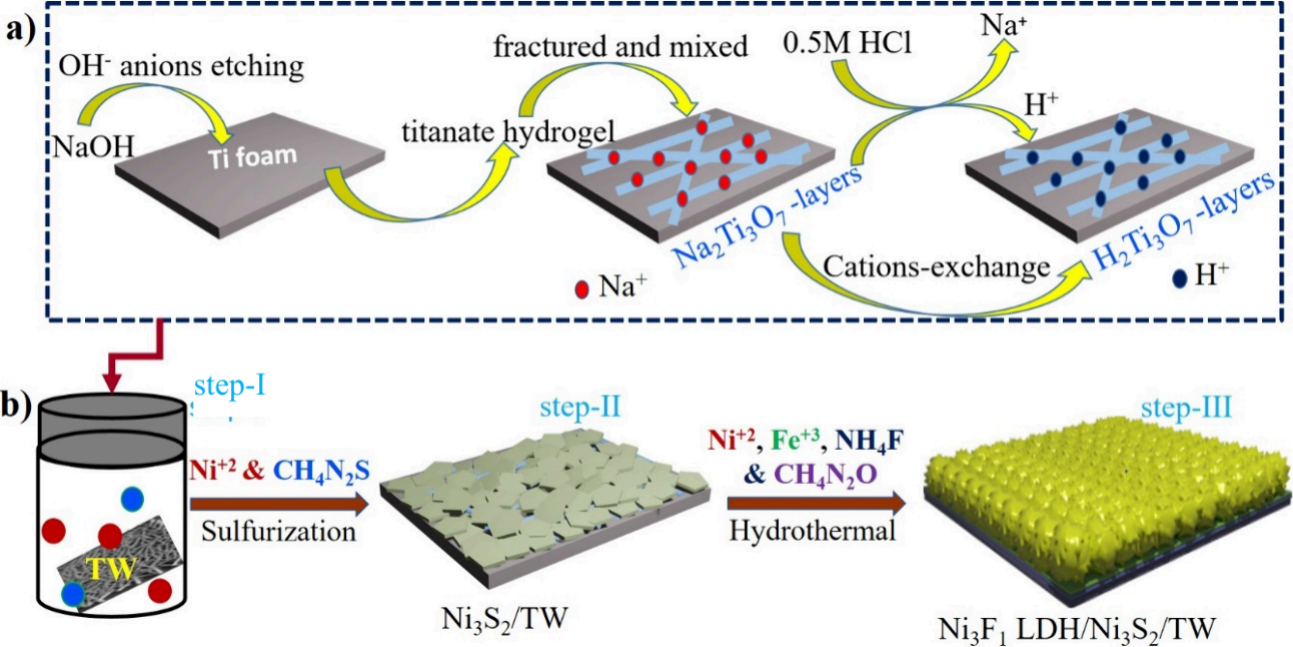
Schematic illustration for (a) titanate web layer formation mechanisms during hydrothermal processing via Ti foam surface modification. (b) Fabrication route of the Ni_3_Fe_1_ LDH/Ni_3_S_2_/TW heterostructure electrocatalyst.

Figures 1a and 1b display a schematically designed formation process to produce titanate web layers and heterostructured Ni_3_Fe_1_ LDH/Ni_3_S_2_/TW electrocatalysts via a hydrothermal process. The Ti foam was initially subjected to OH^–^ anions in NaOH to generate a titanate hydrogel, which was later fractured and mixed to form a layer of Na_2_Ti_3_O_7_ on the TF. The H_2_Ti_3_O_7_ (TW) nanostructure was generated after immersing the Na_2_Ti_3_O_7_ web into 0.5 M HCl, which allows a thermodynamically favorable cation-exchange process between Na^+^ and H^+^ ions. Based on the XRD result and the expected reactions during the synthesis in the alkaline hydrothermal reaction processes,^31^ the suggested sequence of formation route in the hydrothermal process is presented in Figure 1 and eqs 1 and 2 below.
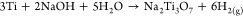
1
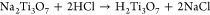
2

Thiourea can be desulfurized (steps I and II) to form Ni_3_S_2_ nanosheets on the titanate web. The two reactions for the formation of Ni_3_S_2_ during hydrothermal treatment are indicated below.^32^ Thiourea hydrolyzed to give HS^–^ ions (eq 3) and reacted with Ni^2+^ to generate Ni_3_S_2_ (eq 4).
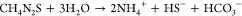
3
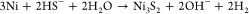
4

The hydroxide ions gradually formed through urea hydrolysis, causing a constant assembly of Ni_3_Fe_1_ LDH on the surface (steps II and III). The possible reactions during formation are shown in eqs 5 and 6 below.
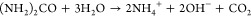
5
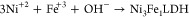
6

### 3.2 Morphology and Structural Characterizations

**Figure 2:**
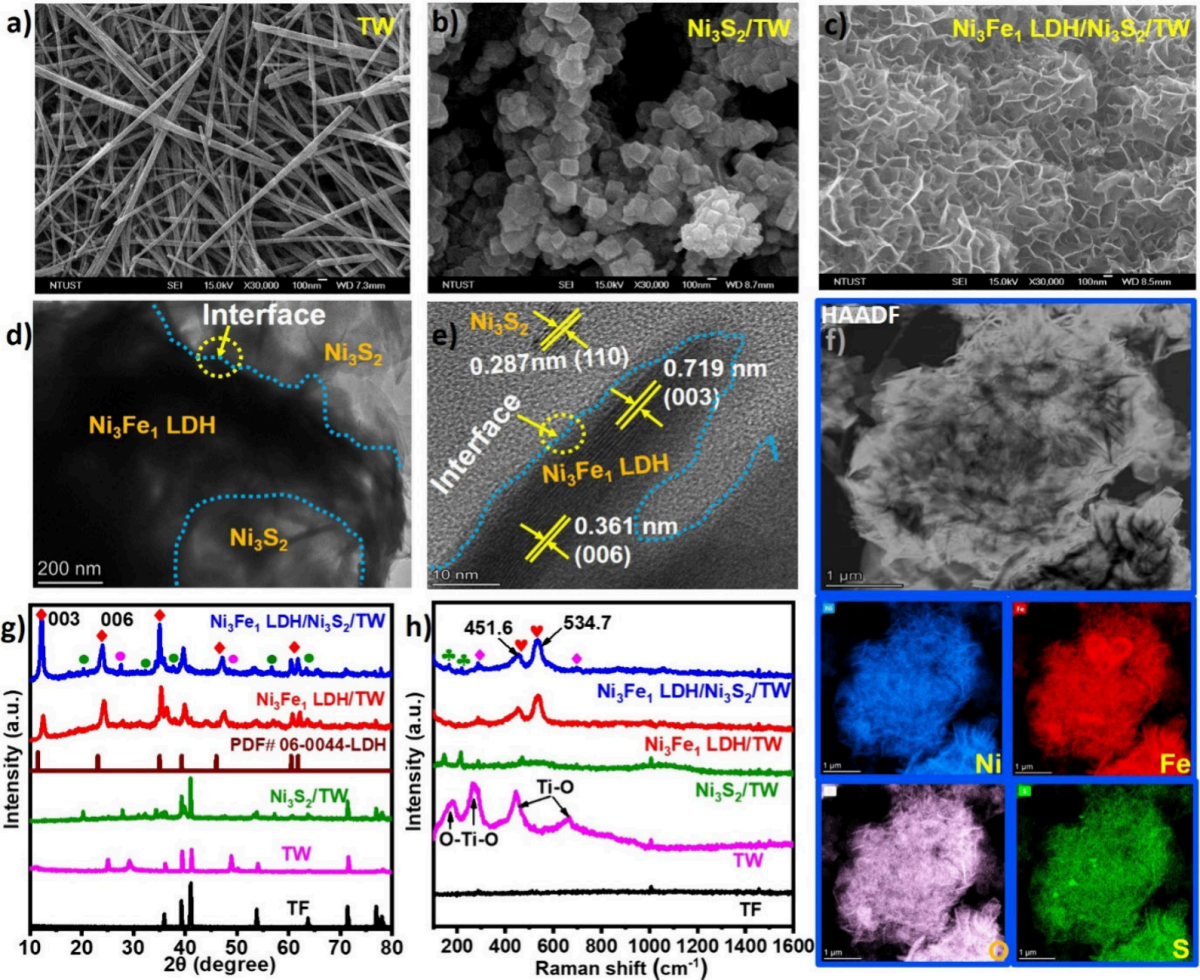
SEM morphology for (a) titanate web layers generated from surface-modified TF, (b) Ni_3_S_2_/TW, and (c) Ni_3_Fe_1_ LDH/Ni_3_S_2_/TW hybrid interface heterostructures. (d) TEM image and (e) HR-TEM image for Ni_3_Fe_1_ LDH/Ni_3_S_2_/TW electrocatalysts. (f) HAADF-STEM image with associated elemental mapping for Ni_3_Fe_1_ LDH/Ni_3_S_2_/TW. (g) XRD patterns for the corresponding electrocatalyst. (h) Raman spectra of the as-synthesized electrocatalysts.

The morphology of electrocatalysts was investigated by using scanning electron microscopy (SEM). Contrary to precleaned TF with a smooth surface, after hydrothermal reaction in an alkaline solution, a web-like titanate layered nanostructure was generated on the surface of TF, as illustrated in Figures 2a and S1a, which is solid evidence for surface modification of Ti foam. The SEM morphology of the N_3_S_2_/TW nanosheet is indicated in Figure 2b. The N_3_S_2_ was formed with an even distribution on TW layers, which was later used as morphology directing for uniform growth of bimetallic Ni_3_Fe_1_ LDH and further forming of the Ni_3_Fe_1_ LDH/Ni_3_S_2_/TW heterostructure (Figure 2c). It displays a three-dimensional interconnected nanoflower open heterostructure, which is advantageous for generating increased electrochemical surface area (ECSA), namely more available and exposed active sites. This indicates that the in situ formed titanate web layers on surface-modified TF create strong interactions with Ni_3_S_2_, further enhancing the uniform dispersion of surface metal LDHs. The Ni_3_S_2_ and N_3_Fe_1_ LDHs were also developed on the unmodified TF to see the advantages of the surface-modified TF. Both cases showed clusters of aggregated structures (Figures S1b and S1c). This signifies the importance of surface modification of the substrate for uniform dispersion and strong interaction among the composites, further enhancing long-term stability. The results obtained from the SEM image of the post-OER electrocatalyst in Figure S1d confirm that the original 3D nanoflower heterostructure remains unaffected after the stability test, suggesting the importance of in situ surface modification. The high-resolution transmission electron microscopy (HR-TEM) image in Figure 2d shows a dense heterointerface of the Ni_3_Fe_1_ LDH/N_3_S_2_/TW structure. The lattice fringe spacings of 0.719 and 0.361 nm were observed, which correspond to the (003) and (006) diffraction planes of the Ni_3_Fe_1_ LDHs, and the crystal lattice spacing of 0.287 nm is ascribed to the (110) plane of Ni_3_S_2_ (Figure 2e). Moreover, HR-TEM images of the Ni_3_Fe_1_ LDH/Ni_3_S_2_/TW electrocatalyst show the interface heterostructure. The blue frames illustrate an interface region, revealing the formation of the Ni_3_Fe_1_ LDH/Ni_3_S_2_/TW interface heterostructure. The HAADF-STEM image shows a nanoflower structure, and elemental mapping displays the uniformly distributed Fe, Ni, O, and S elements (Figure 2f).

X-ray diffraction (XRD) was applied to examine the crystalline and phase structures. As observed in Figure 2g, the bare TF and TW layers have peaks at similar 2θ values, except differing with peaks formed on TW at 25.1 and 48.9, confirming the formation of a titanate web with anatase type matching with the reference (PDF 36-0654) (Figure S2a), and impurities of the rutile phase at 27.9. For the Ni_3_Fe_1_ LDH/Ni_3_S_2_/TW electrocatalyst, characteristic peaks at 2θ = 12.1, 23.8, 34.8, 39.5, 47.1, 60.5, and 61.8 are indexed to the (003), (006), (012), (015), (018), (110), and (113) planes of hexagonal Ni_3_Fe_1_ LDH phases, respectively (PDF 06-0044). This appeared in accordance with the previously reported diffraction peaks of the NiFe LDH hexagonal crystal phase.^9^ The peaks of Ni_3_S_2_ at 21.60, 31.90, 38.60, and 56.30 are consistent with the (101), (110), (003), and (300) planes of Ni_3_S_2_.^33^ The XRD patterns of the Ni_3_Fe_1_ LDH/Ni_3_S_2_/TW heterostructure in Figure S2b were indexed. After Ni_3_S_2_ was integrated, there was a slight shift to a lower angle at 2θ (2θ = 12.1, 23.8) of Ni_3_Fe_1_ LDH at the (003) and (006) planes in Ni_3_Fe_1_ LDH/Ni_3_S_2_/TW, suggesting an expansion of interlayer basal spacing. Furthermore, the peak intensity becomes sharper and increases, which might indicate an enhanced degree of crystallinity of the electrocatalyst. Further, based on the estimation using Scherer’s equation, the LDHs’ crystalline size has decreased from 15.86 to 14.43 nm, as indicated in Table S2. The small size of surface LDH will promote improved OH^–^/O_2_ transport across the surface–interface region and lead to more accessible sites,^34^ enhancing the OER/HER electrocatalyst activities.

Raman spectroscopy was used to validate the formation and phase compositions of the electrocatalyst. As indicated in Figure 2h, the broad peaks around 168 and 216.4 cm^–1^ were ascribed to Ni_3_S_2_, agreeing with XRD findings. The prominent peaks at 455 and 534.7 cm^–1^ ensure the effectively created Ni_3_Fe_1_ LDHs over the Ni_3_S_2_/TW, which were correlated to the symmetric vibration of Ni–OH and Ni–O, signifying the construction of Ni(OH)_2_.^35^ The Raman shift for TW shows peaks between 200 and 600 cm^–1^ matching Ti–O bonds, showing the creation of titanate web layers on the surface of TF. The intercalated anions in the LDH interlayers have been verified by using FT-IR spectroscopy (Figure S2c). The OH^–^ bond vibration mode in the hydroxide layer was identified at broad bands of 3440 and 1636 cm^–1^, OH^–^ associated with adsorbed water at the surface and the interlayer of LDHs. The carbonate anion of the asymmetric stretching mode existed at about 1385 cm^–1^, and the vibration mode from 651 to 550 cm^–1^ belongs to the stretching of O–M–O and M-O in the electrocatalyst.

**Figure 3:**
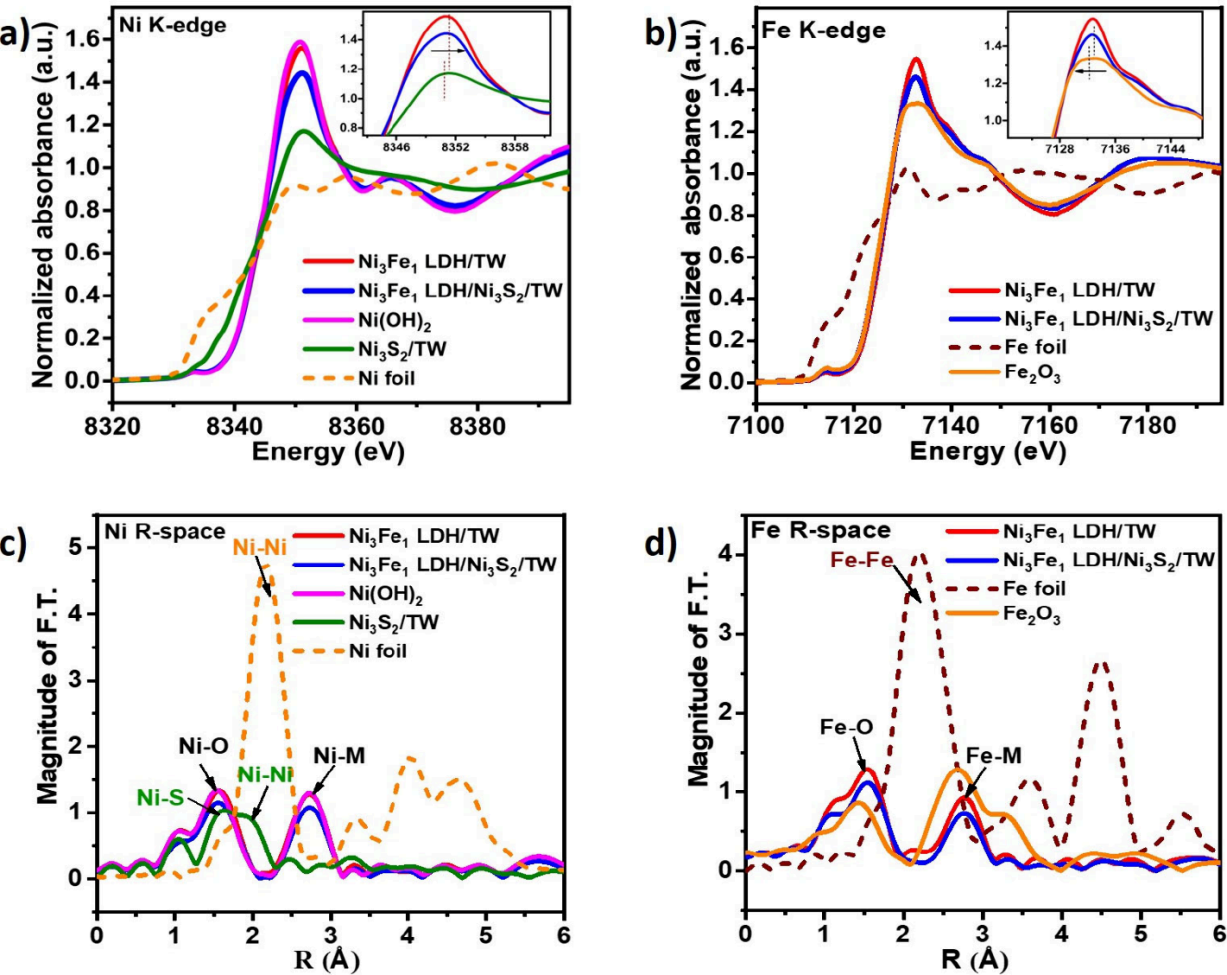
Local structure characterization of the electrocatalysts. The XANES spectra for the (a) Ni K-edge and (b) Fe K-edge of as-prepared electrocatalysts. The EXAFS spectra for the (c) Ni and (d) Fe R-space for the respective electrocatalysts.

The X-ray absorption spectroscopy (XAS) was employed to reveal the coordination number, the local symmetry, the valence state, and the electronic interaction in the heterostructure among the components. The position and intensity of the white line absorption can determine the oxidation state and electronic interaction. The change in the white-line intensity is due to an electronic effect, likely an interfacial electron transfer caused by an electronic interaction at interfaces.^36^ As indicated in Figure 3a, the X-ray absorption near edge structure (XANES) spectra for the Ni K-edge exhibit a pre-edge shoulder at about 8332.8 eV, ascribed to the shift of electron (1s → 3d) orbitals. The absorption peak of the Ni K-edge existed at 8350.8 eV, resulting from a dipole electronic change (1s → 4p) in orbitals.^37^ The main absorption peak of the Ni K-edge in the Ni_3_Fe_1_ LDH/Ni_3_S_2_/TW heterostructure shifted to higher binding energy with reduced intensity when compared with the pristine Ni_3_Fe_1_ LDH/TW (Figure 3a and inset), demonstrating a change in the electronic structure of Ni species in the Ni_3_Fe_1_ LDH/Ni_3_S_2_/TW heterostructure. The electronic interaction of the Ni_3_Fe_1_ LDH and Ni_3_S_2_ interface also agrees with the shift observed from the XPS data. The binding energy of the Fe K-edge absorption peak slightly shifted to a higher position (Figure 3b, inset), compared to Ni_3_Fe_1_ LDH/TW and Fe_2_O_3_, along with a decrease in the white line intensity relative to Ni_3_Fe_1_ LDH/TW, suggesting an electronic interaction and alteration in the electronic properties of Fe in the Ni_3_Fe_1_ LDH/Ni_3_S_2_/TW electrocatalyst due to the heterostructure.^34^ This coupling effect controls the redox properties of Ni and Fe metal ions, triggering active phases during the OER.

**Figure 4:**
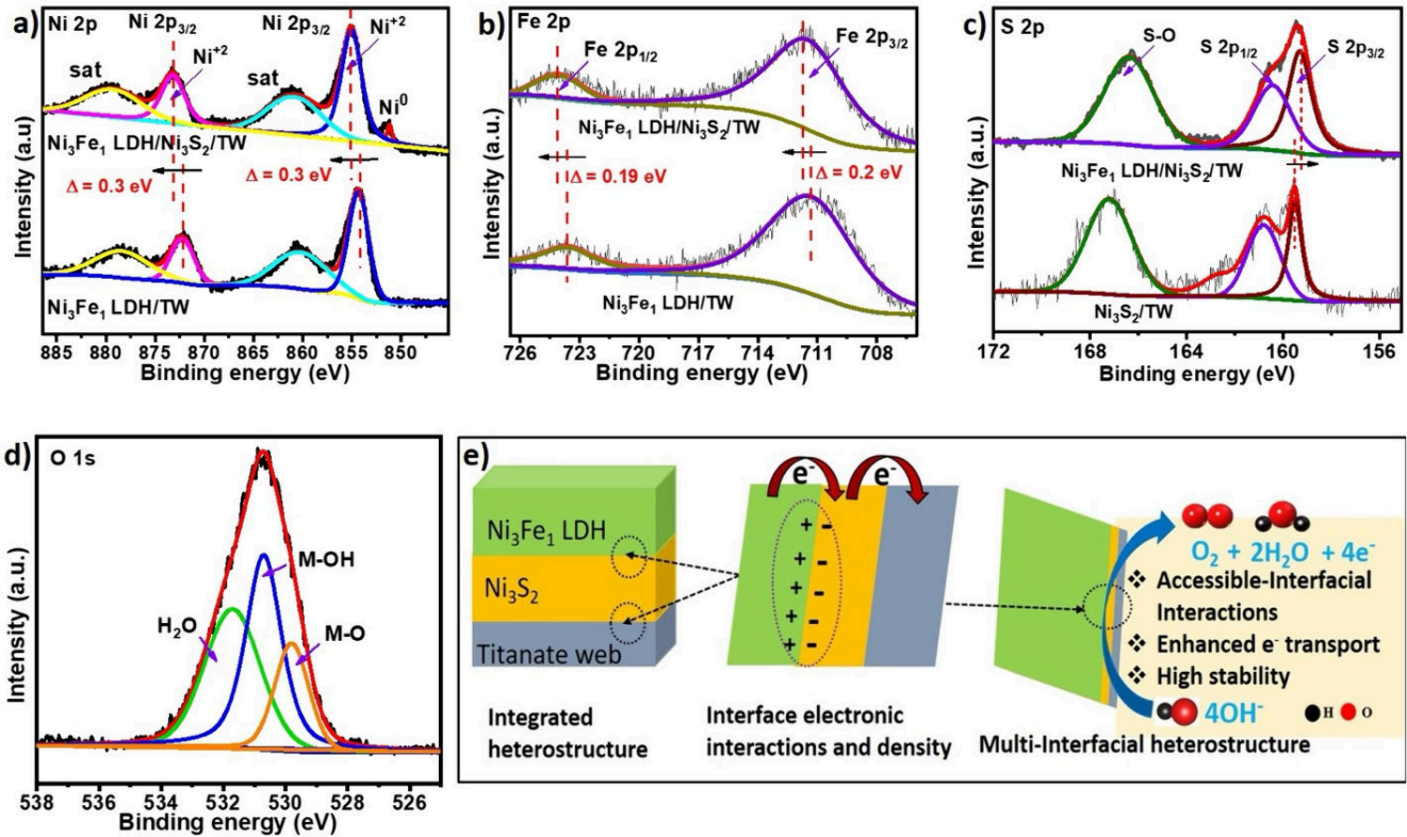
Surface characterizations of the as-prepared electrocatalysts. XPS spectra of (a) Ni 2p and (b) Fe 2p for Ni_3_Fe_1_ LDH/TW and Ni_3_Fe_1_ LDH/Ni_3_S_2_/TW. (c) XPS spectra of S 2p for Ni_3_S_2_/TW and Ni_3_Fe_1_ LDH/Ni_3_S_2_/TW. (d) XPS spectra of O 1s for Ni_3_Fe_1_ LDH/Ni_3_S_2_/TW. (e) Scheme of the electronic interactions at the heterostructure interfaces.

The local structural characteristics of the Ni and Fe in Ni(OH)_2_, Ni_3_Fe_1_ LDH/TW, and the Ni_3_Fe_1_ LDH/Ni_3_S_2_/TW heterostructure were determined using the EXAFS fitting, as presented in Table S1 and Figures S3a–S3d. The EXAFS spectra for the Ni and Fe K-edges in Ni_3_Fe_1_ LDH/TW and Ni_3_Fe_1_ LDH/Ni_3_S_2_/TW exhibit two primary peaks that are attributed to metal–metal (M–M) and metal–oxygen (M–O) bonds (Figures 3c and 3d). The EXAFS fitting results for Ni in the Ni_3_Fe_1_ LDH/Ni_3_S_2_/TW heterostructure have a lower bond length (Ni–O = 2.03 Å) and coordination number (*N* = 4.7) compared to Ni(OH)_2_ (2.04 Å, *N* = 6.0) and Ni_3_Fe_1_ LDH (2.04 Å, *N* = 5.5), indicating the decline in coordination near the Ni–O and Ni–M sites, which leads to the creation of distortion, which in turn contributes to the reduction in size. Interestingly, the EXAFS spectra of Ni_3_S_2_/TW in Figure 3c show two peaks of intensity at 1.61 and 1.99 Å assigned to Ni–S and Ni–Ni bonds, confirming the formation of Ni–S and a network of Ni–Ni bonds matching Ni–Ni bonds in the Ni foil. According to the Fe K-edge EXAFS spectra (Figure 3d), Fe–O and Fe–M intensity in the Ni_3_Fe_1_ LDH/Ni_3_S_2_/TW heterostructure is lower than in Ni_3_Fe_1_ LDHs. This might be due to reduced coordination environments and disordered structures near Fe sites, as calculated by the fitting parameters in Table S1. The reduced intensity indicates structural distortion due to interface-engineered Ni_3_S_2_ nanosheets. The disordered structure with reduced coordination environments boosts intrinsic site activity and populations, increasing electronic conductivity and the ease of intermediate adsorption on the catalyst. Further on, the binding energy of the Ti K-edge decreased in Ni_3_S_2_/TW compared to TW (Figure S3e, insets). This implies that the oxidation state of Ti in Ni_3_S_2_/TW was reduced, confirming electron transfer from Ni_3_S_2_ to TW. However, the binding energy of the Ti K-edge spectra of the Ni_3_Fe_1_ LDH/Ni_3_S_2_/TW electrodes exists between TW and Ni_3_S_2_/TW. Figure S3f shows EXAFS spectra peaks of the Ti 2p R-space in TW, N_i3_S_2_/TW, and Ni_3_Fe_1_ LDH/Ni_3_S_2_/TW, indicating Ti–Ti and Ti–O bond formation in the interface-engineered heterostructure.

X-ray photon spectroscopy (XPS) was measured to study the interfacial electronic interaction and the chemical state of atoms. The XPS survey spectrum of the as-synthesized Ni_3_Fe_1_ LDH/Ni_3_S_2_/TW heterostructure (Figure S4a) validates that the Fe, Ni, S, and O atoms are present in the samples. The Ni 2p in Ni_3_Fe_1_ LDH/Ni_3_S_2_/TW (Figure 4a) exhibits two peaks at 872.3 and 854.6 eV consistent with Ni 2p_1/2_ and Ni 2p_3/2_, indicating the existence of Ni^2+^.^38^ When compared to the Ni 2p of Ni_3_Fe_1_ LDH/TW at 872 and 854.3 eV, it shifts to a higher binding energy by 0.3 eV, indicating the presence of electron transport at the heterojunction interfaces, further confirming the strong electronic interactions in the Ni_3_Fe_1_ LDH/Ni_3_S_2_/TW composites.^18^ However, compared to Ni_3_S_2_/TW peaks occurring at 872.5 and 854.7 eV (Figure S4b), the binding energy of Ni 2p in the multi-interface heterostructure is shifted negatively by 0.2 and 0.1 eV, suggesting strong electronic interactions and the existence of more electron density at the interface as a result of electron transfer from Ni_3_Fe_1_ LDHs to Ni_3_S_2_ sites. A small peak at 852.1 eV was also identified, which corresponds to the typical peak of Ni^0^ indexed to a network of metallic Ni–Ni bonds in Ni_3_S_2_. This is further evidence for the formation of an interface hybrid heterostructure, as a similar peak also appeared for Ni_3_S_2_/TW (Figure S4b). The Fe 2p spectrum of Ni_3_Fe_1_ LDH/Ni_3_S_2_/TW (Figure 4b) shows two typical peaks at 723.7 and 711.5 eV attributed to Fe 2p_1/2_ and Fe 2p_3/2_, respectively.^8^ When compared to the binding energy of pristine Ni_3_Fe_1_ LDH/TW at 723.5 and 711.31 eV, the Fe 2p peaks are shifted toward higher binding energies by 0.2 and 0.19 eV, indicating robust electronic interactions due to the transfer of electrons at the interface regions of the heterostructure. Moreover, evidence of electronic interactions is provided by a comparison of the S 2p spectra in the Ni_3_S_2_ and Ni_3_Fe_1_ LDH/Ni_3_S_2_/TW, which can be deconvoluted into three peaks at 160.2, 162, and 167.2 eV representing S–S, Ni–S, and S–O, respectively (Figure 4c), confirming the existence of S^2–^.^8^ The S 2p peaks in the Ni_3_Fe_1_ LDH/Ni_3_S_2_/TW are moved to lower binding energy than those in the Ni_3_S_2_/TW. This indicates an increase in the electron population at the interface site near S atoms, indicating electron migration from the bimetallic LDH surface to the Ni_3_S_2_ interface boundaries. The peak at 162.9 eV reveals the satellite formation.^1^ Furthermore, the O 1s spectra deconvoluted into three peaks characteristic at 529.70, 530.8, and 531.7 eV (Figure 4d), showing M–O, M–OH, and O–H of adsorbed H_2_O, respectively.^39^ In addition, to see the electronic interactions among the components of the heterostructure, we checked the XPS spectra of Ti 2p in the TW (Figure S4c). The Ti 2p peaks found at 457.6 and 463.5 eV refer to Ti 2p_3/2_ and 2p_1/2_. The 2p peaks for Ti in Ni_3_S_2_/TW appeared at 457.1 and 463.3 eV, showing reduced binding energies by 0.5 and 0.2 eV, respectively. This suggests that the Ti^4+^ has an electron-withdrawing impact in the strong metal–support interactions favoring electronic migration, further enhancing conductivity and stability. The binding energy of Ti 2p also decreased in the Ni_3_Fe_1_ LDH/Ni_3_S_2_/TW heterostructure as compared to TW and slightly increased compared to Ni_3_S_2_/TW. Therefore, as indicated in Figure 4e, the strong interfacial electronic interactions are exhibited in the multi-interface heterostructure that can be responsible for fast charge transfer with the modulation of electronic conductivity, enhanced intrinsic activity, and long-term stability

### 3.3 Electrocatalytic OER Performance Evaluation

**Figure 5:**
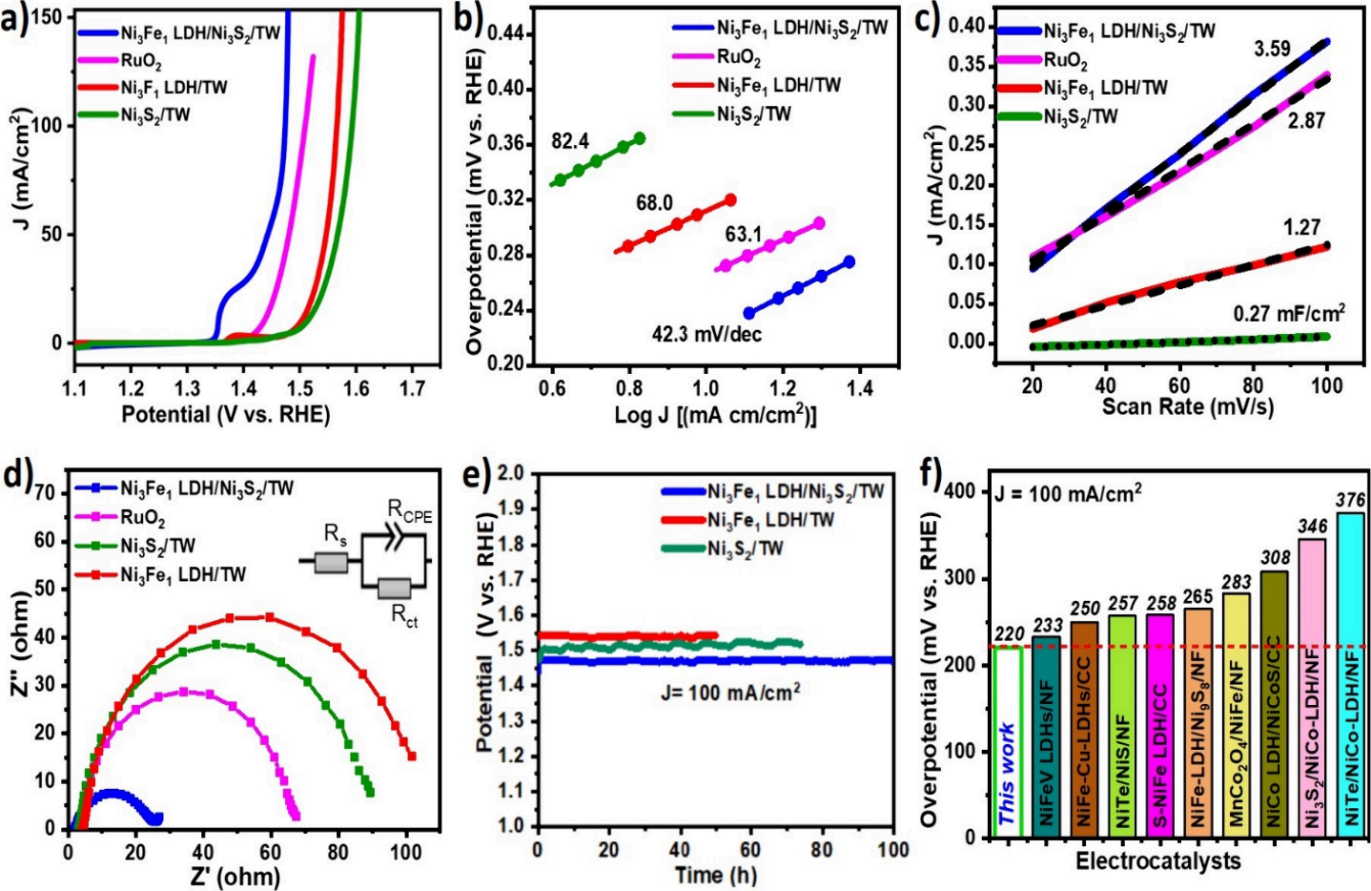
(a) OER LSV polarization curve of electrocatalysts in 1 M KOH at a scan rate of 1 mV/s. (b) The respective Tafel plots of the electrocatalysts. (c) Electrochemical capacitive currents vs scan rate of the samples. (d) Nyquist plots for EIS with its related electrocatalysts. (e) Chronopotentiometric stability curves for Ni_3_Fe_1_ LDH/Ni_3_S_2_/TW, Ni_3_Fe_1_ LDH/TW, and Ni_3_S_2_/TW at a constant current density of 100 mA cm^–2^. (f) Comparison of the overpotentials at 100 mA cm^–2^ for Ni_3_Fe_1_ LDH/Ni_3_S_2_/TW with those of reported electrocatalysts.

The OER performance of as-synthesized electrocatalysts was assessed using a three-electrode setup in 1 M KOH. As verified in the linear sweep voltammetry (LSV) curves (Figure 5a), the Ni_3_Fe_1_ LDH/Ni_3_S_2_/TW heterostructure interface needs a smaller overpotential of 220 mV at 100 mA cm^–2^, as compared to Ni_3_S_2_/TW (350 mV), Ni_3_Fe_1_ LDH/TW (330 mV), and commercial RuO_2_ (270 mV). The earliest redox onset potential reflects the conversion of Ni(OH)_2_ to NiOOH species, which increased the intrinsic site activity. The OER mechanisms in alkaline conditions are generally indicated by eqs 7–10.^40^
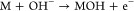
7
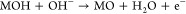
8
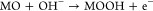
9
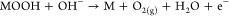
10

The kinetics of the OER electrocatalytic mechanism were evaluated through the Tafel slope. As indicated in Figure 5b, the Ni_3_Fe_1_ LDH/Ni_3_S_2_/TW electrocatalyst exhibited a smaller Tafel slope of 42.3 mV dec^–1^ than the pristine Ni_3_Fe_1_ LDH/TW (68 mV dec^–1^) and Ni_3_S_2_/TW (82.4 mV dec^–1^), demonstrating the best response to the OER kinetics due to quick electron transfer. Furthermore, to realize the cause of superb OER and better understand the intrinsic electrocatalytic properties of the catalysts, the electrocatalytic ECSA was measured. It provides information about the number of electrochemically active sites on the electrodes, and therefore it is important to evaluate the site populations or the active site exposure. Accordingly, the double-layer capacitance (*C*_dl_) was evaluated using cyclic voltammetry (CV) tests in the nonfaradaic region (Figures 5c and S5a–S5d) to assess the corresponding ECSA based on the positive correlation between *C*_dl_ and ECSA. Then, ECSA was computed by eq S1, where *C*_s_ is the specific capacitance of the flat surface (40 μF cm^–2^) in 1 M alkaline solution.^41^ The Ni_3_Fe_1_ LDH/Ni_3_S_2_/TW electrocatalyst retains the largest *C*_dl_ with greater ECSA (89.8 cm^2^) compared to pristine Ni_3_Fe_1_ LDH/TW (31.5 cm^2^) and Ni_3_S_2_ (6.8 cm^2^) (Table S2). A high ECSA value for the Ni_3_Fe_1_ LDH/Ni_3_S_2_/TW electrocatalyst indicates high active sites available on the surface of electrocatalysts.^34^ Furthermore, the site activity (SA) refers to the specific catalytic activity on the exposed surface of the catalyst at a specific constant potential, which is evaluated as the current per electrochemically active surface area of the electrocatalyst^42^ and evaluated according to the previous report^7^ based on eq S2. The site activity for the Ni_3_Fe_1_ LDH/Ni_3_S_2_/TW heterostructure electrocatalyst is too high, being 4.4 and 1.4 times higher than that of Ni_3_Fe_1_ LDH/TW and Ni_3_S_2_/TW, respectively, as indicated in Table S2, which reflects the enhanced performance of the OER inducing the earliest onset potential, as can be seen in the LSV polarization curves. These results confirm that the integrated Ni_3_Fe_1_ LDH/Ni_3_S_2_/TW heterostructure efficiently increases the intrinsic catalytic site activity and boosts electrocatalytic OER performances. To examine the electron transport capability and kinetics of the electrocatalysts at the electrode/electrolyte interfaces, electrochemical impedance spectroscopy (EIS) was measured from 100 kHz to 0.01 Hz at an anodic polarization potential of 1.5 V. As observed in Nyquist plots for EIS in Figure 5d, the smaller charge transfer resistance value (24.8 Ω) for the Ni_3_Fe_1_ LDH/Ni_3_S_2_/TW heterostructure, significantly smaller than RuO_2_ (68.1 Ω), Ni_3_S_2_/TW (89 Ω), and Ni_3_Fe_1_ LDH/TW (102.5 Ω), indicates that excellent interfacial electronic interactions were created at the heterostructured interfaces, contributing to improved electrical conductivity and boosting the OER catalytic kinetics.^43^ The Ni_3_S_2_/TW has greater charge transfer capability than the Ni_3_Fe_1_ LDH/TW electrocatalyst due to the rich network of Ni–Ni bonds that creates intrinsic metallic behavior. The stability of the electrocatalyst was assessed by the chronopotentiometry test (Figure 5e). It demonstrated remarkable stability over 100 h at an overpotential of 220 mV. The great stability of the Ni_3_Fe_1_ LDH/Ni_3_S_2_/TW electrocatalyst was also confirmed by the LSV polarization curves of the pre- and post-chronopotentiometric stability test (Figure S5e), showing insignificant changes. Moreover, the stability of the Ni_3_Fe_1_ LDH/TW and Ni_3_S_2_/TW electrocatalysts was checked to observe their contribution to the stability. The Ni_3_S_2_/TW offers significant stability and is greater than the Ni_3_Fe_1_ LDH/TW. Thus, it was observed that the electronic effects and close interactions between LDHs, Ni_3_S_2_, and TW stabilize the electrocatalyst, resulting in long-term stability operations. Likewise, compared with the related literature, the Ni_3_Fe_1_ LDH/Ni_3_S_2_/TW electrocatalyst activity indicates a small overpotential (Figure 5f). Moreover, Table S3 compares the performances of the Ni_3_Fe_1_ LDH/Ni_3_S_2_/TW heterostructure with previous reports in the literature. The stability and overpotential of different samples are selectively considered and compared with those of the electrocatalysts evaluated at the same applied current density. The Ni_3_Fe_1_ LDH/Ni_3_S_2_/TW electrocatalyst for the OER shows the lowest Tafel slope, small overpotential, and the most stable performances. To examine the operation efficiency of electrons, the faradaic efficiency (FE) was assessed using a H-type cell at a constant current of 100 mA cm^–2^ for 1 h. The FE is computed by associating experimentally produced O_2_ with theoretically generated O_2_ by the water displacement method. As revealed in Figure S5f, the moles of O_2_ gas generated agree well with the theoretical values, and the calculated FE is about 96.1%.

### 3.4 Post-OER Electrocatalytic Characterizations and Mechanistic Insights

**Figure 6:**
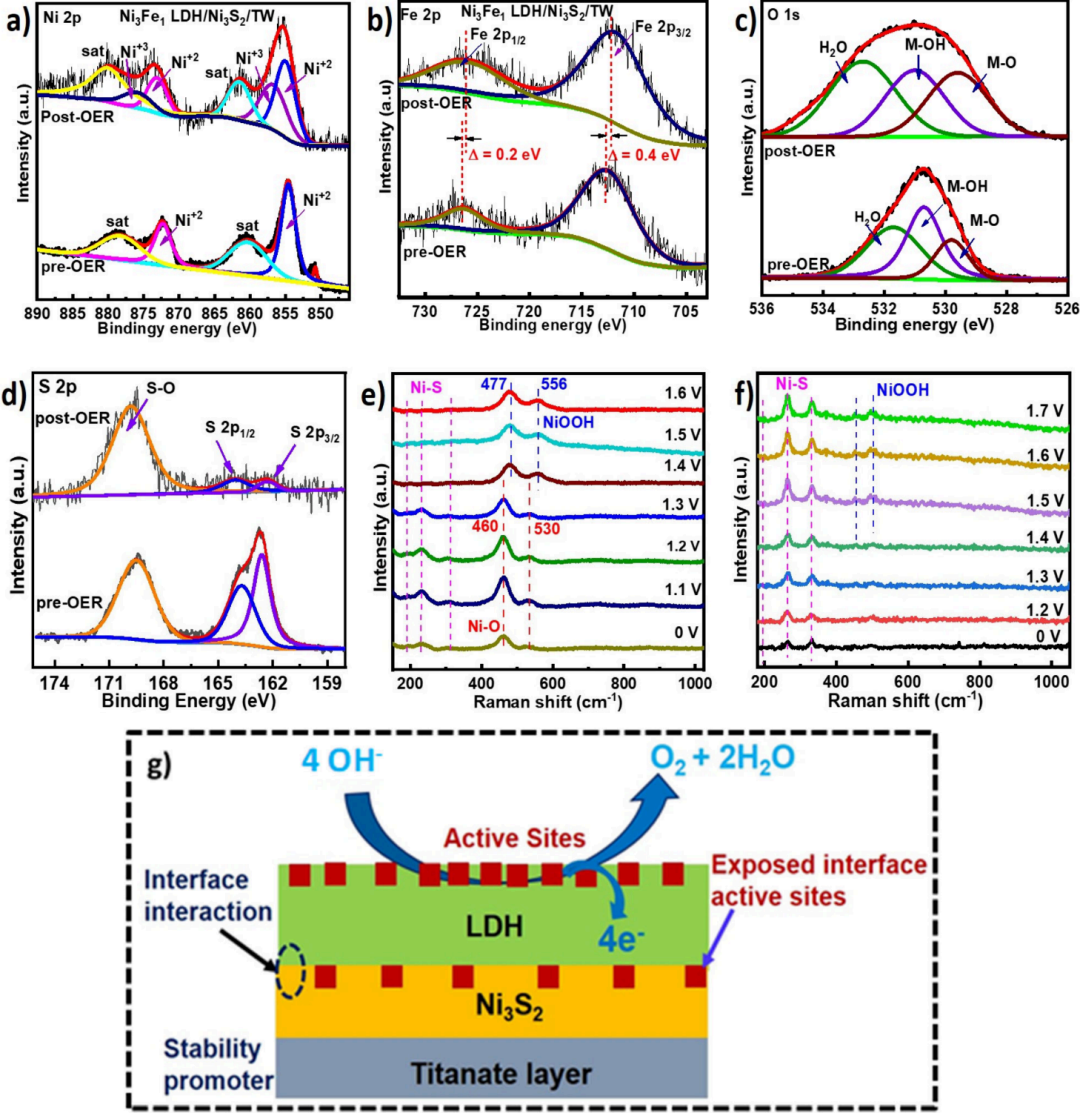
XPS characterizations for comparison of the pre- and post-OER electrocatalysts. XPS spectra of (a) Ni 2p, (b) Fe 2p, (c) O 1s, and (d) S 2p of the as-prepared Ni_3_Fe_1_ LDH/Ni_3_S_2_/TW electrocatalyst. The in situ Raman spectra for (e) Ni_3_Fe_1_ LDH/Ni_3_S_2_/TW and (f) Ni_3_S_2_/TW measured in 1 M KOH solution. (g) Coupled multiple interfacial engineering and proposed mechanistic effects on the heterostructured electrocatalysts.

The electrocatalyst was studied after the OER process to further study the active site and mechanisms, changes in surface chemical states, structural evolution, and stability. The XPS data of the Ni 2p in Ni_3_Fe_1_ LDH/Ni_3_S_2_/TW heterostructure provided two spin–orbit doublets (Figure 6a). The post-OER peaks at a binding energy of 856 and 873.2 eV belong to Ni^3+^, converted from Ni^2+^ into Ni^3+^, generating a NiOOH active phase.^44^ The conversion was facilitated by the strong electronic interaction in the components, which is in agreement with in situ Raman shifts (which will be discussed next). The XPS result in Figure 6b displays pre- and post-OER comparison of Fe 2p for the Ni_3_Fe_1_ LDH/Ni_3_S_2_/TW heterostructure, indicating two peaks matching Fe 2p_1/2_ and Fe 2p_3/2_. The pre-OER binding energy existed at 711.5 and 723.7 eV, while the post-OER binding energy occurred at 711.1 and 723.5 eV, which is dropped by 0.4 and 0.2 eV at both Fe 2p_3/2_ and Fe 2p_1/2_, respectively. The decreased binding energy of Fe during the OER process is attributed to partial charge transfer from the Ni-oxyhydroxides to Fe, which modifies the electronic properties of the Ni. This can further facilitate the redox transition of Ni^2+^/Ni^3+^, which might be the reason for the activity enhancement.^45^ The O 1s XPS spectra peaks at 529.5, 530.8, and 533.3 eV correspond to the M–O, M–OH, and adsorbed water.^46^ The O 1s XPS spectra measurements and the peak area of oxygen intermediates in pre- and post-OER for Ni_3_Fe_1_ LDH/Ni_3_S_2_/TW electrocatalyst were compared in Figure 6c. As indicated in Table S4, the calculated peak area of the M–O ratio was significantly increased after the OER, showing the enhanced formation of the M–O bond at the expense of decreased M–OH ratio, further confirming the generation of NiOOH active species during OER.^47^ The XPS result of S 2p indicates that the M–S bond intensity was decreased (Figure 6d). At the same time, M–O species increase for O 1s in the post-OER electrocatalyst, which might correspond to the transformation of Ni_3_S_2_ to the NiOOH phase, agreeing with in situ Raman spectra of M–S species at certain applied potential as described below. In this case, the extra OER active interface sites are created, where S undergoes a transformation to the S–O species and causes the dynamic reconstruction of the NiOOH active phase formation. The resulting MOOH phase is considered the active species for OER.^48^ The typical S–O peak of Ni_3_S_2_ can still be significantly observed after the OER, indicating the S species may have experienced partial conversion into SO_4_^2–^ ion adsorption, which could substantially improve reactive intermediate adsorption and lower the stepwise energy barrier for the OER and might be used as an anticorrosive by enhancing the stability of the electrocatalyst.^49^ XRD results show that the Ni_3_Fe_1_ LDH/Ni_3_S_2_TW electrocatalyst retains its structure after the OER testing, as seen in Figure S6a. No noticeable new phases were observed, demonstrating good structural stability of the catalysts. Further, to disclose the nature of the active site and screen the interfacial structural evolution of the Ni_3_Fe_1_ LDH/Ni_3_S_2_/TW electrocatalysts during an oxidation time with a range of applied potentials from 1.1 to 1.6 V vs a reversible hydrogen electrode (RHE), in situ Raman spectroscopy was executed in 1 M KOH aqueous solution. As shown in Figure 6e, the two distinct peaks around 460 and 530 cm^–1^ can be seen in the first in situ Raman spectra of Ni_3_Fe_1_ LDH/Ni_3_S_2_/TW, which could be related to Ni^II^–O vibrations in the Ni(OH)_2_. The Raman peaks at 460 and 530 cm^–1^ were blue-shifted to 477 and 556 cm^–1^ with an increase in applied potentials (≥1.4 V), which represent the e_g_ bending vibration of Ni^III^–O and the A_1g_ stretching vibration of Ni^III^–O for nickel oxyhydroxide species, respectively, and indicate that Ni(OH)_2_ was converted into the NiOOH active phase during the OER.^50^ In addition, it can be witnessed that the peaks at ∼192 and ∼232 cm^–1^ are related to the existence of Ni–S species, which gradually decline as the applied potential reaches above 1.4 V, indicating the Ni–S species were also converted into NiOOH active phases. This confirms that the interface sites can promote the NiOOH active species and eventually enhance intermediate adsorption. The weak distinctive peak displayed at 356 cm^–1^ is assigned to the Fe–O stretching modes of Fe^III^–O species.^51^ Most importantly, in situ Raman spectroscopy was examined for Ni_3_S_2_/TW to investigate the role of Ni_3_S_2_ in the creation of an exposed active interface site and the interfacial phase evolution during the reaction (Figure 6f). The peaks observed at 191.8, 261, and 329 cm^–1^ correspond to the Ni–S bonds. New peaks appeared at 456 and 534 cm^–1^ when the applied potential was higher than 1.5 V, related to the conversion of Ni–S species into the NiOOH active phase. This confirms the creation of the NiOOH active phase in the vicinity of the exposed interface region. The in situ Raman spectroscopy for Ni_3_S_2_/TW shows the role of the Ni_3_S_2_ interface in creating an exposed active interfacial site and enhancing electronic interactions of constituents. This eventually enhances intermediate adsorption and produces synergistic effects that increase OER activity. Thus, the in situ Raman analyses reveal Ni is an active species, which agrees with the XPS data of the post-OER findings. The multi-interface-engineered heterostructure with different phase components generally exposes more accessible active sites at the surface and interface sites with improved reaction kinetics and enhanced activity. Thus, based on the post-OER characterization evidence and in situ Raman spectroscopy, the deduced mechanistic effects on the heterostructured electrocatalysts (Figure 6g) and the as-proposed reaction mechanisms are provided in Figure S6b, which indicates the synergistic effects of surface/interface Ni integrating components of Ni_3_Fe_1_ LDHs and Ni_3_S_2_ for the optimization of adsorption–desorption intermediates and for exposing more accessible active sites through strong electronic interactions during the OER reaction. Therefore, the synergy leads to significantly improved Ni_3_Fe_1_ LDH/Ni_3_S_2_/TW heterostructure electrocatalyst OER activity and long-term stability.

### 3.5 Electrocatalytic HER and Overall Water Splitting Performance

**Figure 7:**
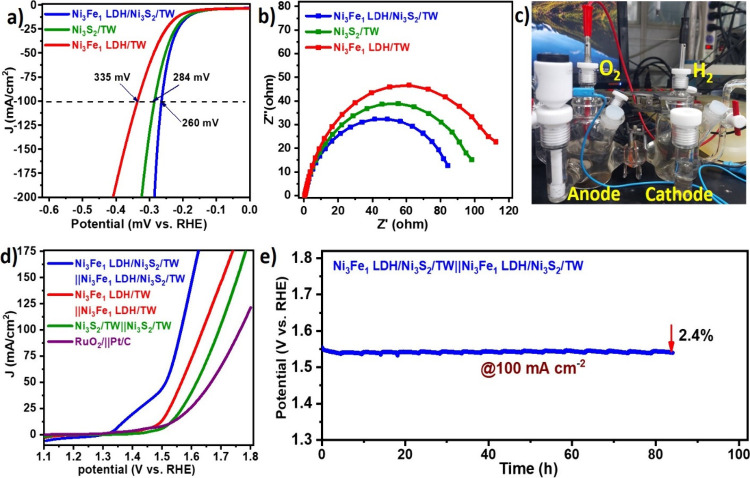
Electrochemical HER overall water splitting performance measurements. (a) Polarization curve of different samples for HER in a 1.0 M KOH solution. (b) Nyquist plots of as-synthesized electrocatalysts. (c) Diagram of the overall water splitting cell system with 1.55 V. (d) Polarization curve comparison for the Ni_3_Fe_1_ LDH/Ni_3_S_2_/TW||Ni_3_Fe_1_ LDH/Ni_3_S_2_/TW with other catalysts using H-type cell two-electrode systems for overall water splitting in 1 M KOH solution. (e) Stability test for the Ni_3_Fe_1_ LDH/Ni_3_S_2_/TW electrocatalyst for the overall water splitting system.

Before building the entire water splitting cell, we measured the LSV of the as-prepared Ni_3_Fe_1_ LDH/Ni_3_S_2_/TW electrocatalysts to assess their HER activity. The lowest overpotential of 260 mV at 100 mA cm^–2^ was observed for the Ni_3_Fe_1_ LDH/Ni_3_S_2_/TW heterostructure compared to others (Figure 7a). Further, to elucidate the superior HER activity of electrocatalysts, the electron transfer kinetics of the Ni_3_Fe_1_ LDH/Ni_3_S_2_/TW, Ni_3_Fe_1_ LDH/TW, and Ni_3_S_2_/TW electrocatalysts were examined using EIS at 0.26 V (Figure 7b). The Ni_3_Fe_1_ LDH/Ni_3_S_2_/TW self-supported electrocatalyst shows excellent charge transfer during the HER path between the electrodes and the electrolyte interfaces, as realized by semicircle diameters smaller than those of the others. Inspired by the high catalytic activities of the self-supported Ni_3_Fe_1_ LDH/Ni_3_S_2_/TW electrocatalyst for both the OER and HER toward alkaline water, a two-electrode water splitting cell was assembled to demonstrate its practical applications, where the Ni_3_Fe_1_ LDH/Ni_3_S_2_/TW electrocatalyst is used for both the anode (OER) and cathode (HER). Hence, the electrocatalysis cell is expressed as Ni_3_Fe_1_ LDH/Ni_3_S_2_/TW||Ni_3_Fe_1_ LDH/Ni_3_S_2_/TW, and so are other samples. Figure 7c shows a digital photograph of the electrolytic cell during the operations. As can be seen in the polarization curves, the Ni_3_Fe_1_ LDH/Ni_3_S_2_/TW electrocatalyst showed better overall water splitting functionality with a cell voltage of 1.56 V relative to the cells assembled by Ni_3_Fe_1_ LDH/TW||Ni_3_Fe_1_ LDH/TW (1.63 V), Ni_3_S_2_/TW||Ni_3_S_2_/TW (1.7 V), and RuO_2_||Pt/C (1.76 V) in 1.0 M KOH (Figure 7d). The electrolysis potential does not change over the 85 h test (Figure 7e), demonstrating remarkable long-term stability during the overall water splitting. Thus, due to its high activity and long-term stability, the integrated Ni_3_Fe_1_ LDH/Ni_3_S_2_/TW electrocatalyst exhibits exceptional features for practical water splitting applications.

### 3.6 Electrocatalytic Evaluation in the Simulated Seawater

Encouraged by the substantial electrocatalytic performances and long-term stability of the Ni_3_Fe_1_ LDH/Ni_3_S_2_/TW heterostructure in alkaline medium, the OER activity and stability were assessed in simulated seawater (1 M KOH + 0.5 M NaCl). The high concentration of chlorine ions, which can generate chlorine (Cl_2_) and hypochlorite (ClO^–^) at the anode, is directed to the competing Cl_2_ chemistry and causes corrosion and poisoning of electrocatalysts.^52^ Alkaline conditions (pH > 7.5) frequently result in an OER potential window (η < 480 mV) as higher potentials are required for the conversion of Cl^–^ anions to ClO^–^. Jung et al.^53^ synthesized an S-doped NiFe LDH on carbon cloth using 1 M KOH + 0.5 M NaCl, which demands an overpotential of 296 mV at a current density of 100 mA cm^–2^. Although it shows good activity in the simulated seawater, it is only stable for 12 h at 100 mA cm^2^ due to weak catalyst–support interactions and an easily corroded carbon-based support. The OER performance of the as-synthesized Ni_3_Fe_1_ LDH/Ni_3_S_2_/TW interface heterostructure (Figure S7a) exhibits considerable activity with an overpotential of 370 mV at a current density of 100 mA cm^–2^. As manifested in Figure S7b, the stability of the electrocatalyst was assessed by a chronopotentiometry test and remained stable for 100 h at 100 mA cm^–2^, indicating pronounced anticorrosive properties due to the Ti-based support materials and Ni_3_S_2_ interface, figuring out the OER selectivity and stability of the electrocatalyst. The S^2–^ ions in the Ni_3_S_2_ provide a negatively charged anion surface, which can efficiently reject Cl^–^ in saltwater and enhance the anticorrosion properties of materials. A simulated seawater environment was also used to assess the behavior of corrosion resistance and the impact of Ni_3_S_2_ on the electrocatalysts (Figure S7c). As compared to the Ni_3_Fe_1_ LDH/TW (0.93 V) and Ni_3_S_2_/TW (1.08 mV), the Ni_3_Fe_1_ LDH/Ni_3_S_2_/TW heterostructure exhibits a positive shift in corrosion potential at 1.11 V. Interestingly, the corrosion potential of Ni_3_S_2_/TW was greater than that of Ni_3_Fe_1_ LDH/TW, suggesting the contribution of Ni_3_S_2_ to enhanced stability by effectively suppressing chloride-induced corrosion in the multiheterointerface components. The LSV curves before and after the OER chronopotentiometric test also confirmed the electrode’s stability, indicating insignificant changes in the post-OER activity (Figure S7d). These findings show that titanate web layers with Ti-based metal substrates can effectively shield against electrochemical corrosion, providing excellent stability under harsh conditions.

## 4 Conclusions

In conclusion, a self-supported multi-interfacial engineered Ni_3_Fe_1_ LDH/Ni_3_S_2_/TW heterostructure was designed on the in situ generated titanate web layers using bimetallic Ni_3_Fe_1_ LDH and Ni_3_S_2_ nanosheets. The designed heterostructure featured outstanding OER activities at a very low overpotential of 220 mV at 100 mA cm^–2^ with a small Tafel slope of 43.2 mV dec^–1^ and excellent stability for 100 h of operation. Numerous aspects account for the superior performance, such as the modulated electronic configuration, exposed abundant interfacial active sites with enhanced kinetics, and optimized free energy for intermediate adsorption–desorption during the OER. The as-synthesized electrocatalyst shows notable HER activity, achieving a low overpotential of 260 mV at 100 mA cm^–2^. The Ni_3_Fe_1_ LDH/Ni_3_S_2_/TW electrocatalyst also demonstrated a cell voltage of 1.56 V at 100 mV cm^–2^ for the overall water splitting with excellent stability for 85 h. Primarily, the long-term stability of the electrocatalyst was attributed to the generated titanate web, which conveys strong catalyst–support interactions for the interface-engineered heterostructure. Besides, the integrated electrocatalyst performs significant OER activity in simulated seawater with an overpotential of 370 mV to attain 100 mA cm^–2^ and admirable stability for 100 h, realizing virtuous OER selectivity and stability. The strong electronic coupling effects among the composite material provide rich multi-interfacial interaction sites, which effectively induce synergistic effects to accelerate charge transfer for promoting electronic conductivity and reaction kinetics, exposing extra active sites near the interface regions, which could enhance the adsorption of the intermediates and persuade the OER performance. The fabrication strategy for the multi-interface-engineered heterostructure provides a route to construct highly active and stable catalysts in energy storage and conversion.

## Supporting Information Available

Details of reagents, materials characterizations, electrochemical measurements, SEM, FE, FT-IR data, XPS spectra, and Ni and Fe K-edge fitting results and comparative results (PDF)
