# Supplementary material for: Nickel–Iron Layered Double Hydroxides/Nickel Sulfide Heterostructured Electrocatalysts on Surface-Modified Ti Foam for the Oxygen Evolution Reaction
Source: ACS Appl Mater Interfaces. 2024 Sep 12;16(38):50602–13. doi: 10.1021/acsami.4c08215 (PMC11440454; doi:10.1021/acsami.4c08215)
Supplement: Supplementary file 1 — am4c08215_si_001.pdf [file am4c08215_si_001.pdf]

Supporting Information

Nickel-Iron Layered Double Hydroxides/Nickel Sulfide Heterostructured Electrocatalysts on Surface-Modified Ti Foam for Oxygen Evolution Reaction

Habib Gemechu Edao [a, c], Chia-Yu Chang [b, c], Woldesenbet Bafe Dilebo [a, c], Fikiru Temesgen Angerasa [a, c], Endalkachew Asefa Moges [a, c], Yosef Nikodimos [a, c], Chemeda Barasa Guta [a, c], Keseven Lakshmanan [b, c], Jeng-Lung Chen [d], Meng-Che Tsai [b, c] *, Wei-Nien Su,[b, c] *, Bing Joe Hwang [a, c, d] *

[a] *Nano-Electrochemistry Laboratory, Department of Chemical Engineering, National Taiwan University of Science and Technology, Taipei 106, Taiwan.*

[b] *Nano-Electrochemistry Laboratory, Graduate Institute of Applied Science and Technology, National Taiwan University of Science and Technology, Taipei 10607, Taiwan*

[c] *Sustainable Energy Development Center, National Taiwan University of Science and Technology, Taipei 106, Taiwan*

[d] National Synchrotron Radiation Research Center, Hsin-Chu 30076, Taiwan

*Corresponding Authors:

E-mail: bjh@mail.ntust.edu.tw (BJ Hwang);

wsu@mail.ntust.edu.tw (WN Su);

mctsai@mail.ntust.edu.tw (MC Tsai)

Chemicals and Reagents

All chemicals and reagents used in this experiment were of analytical grades. Hydrochloric acid (HCl, 37%), Acetone (C_3H_6O, 99.5%), Ethanol (99.5%), and DI water were used for washing and cleaning purposes. Nickel (II) nitrate hexahydrate ($Ni(NO_3)_2{\cdot}6H_2O$, 99.9%), Iron (III) nitrate nonahydrate ($Fe(NO_3)_3{\cdot}9H_2O$, 99.9%), urea ($CO(NH_2)_2$ > 98%), ammonium fluoride (NH_4F > 98%), thiourea ($CS(NH_2)_2$ > 98%) and potassium hydroxide (KOH, 99.99%) were purchased from Sigma-Aldrich. The Ti foam (TF, thickness of 1.6 mm) was purchased (Sheng Qiang Co., Ltd. Jiangsu, China) and used as a substrate.

Materials Characterizations

The XRD analysis was performed by a Bruker D2 XRD machine outfitted with Cu-K irradiation photon source under (30 kV, λ = 1.5406 Å, 40 kV, and 100 mA) at 2θ range from (10 - 80°). The ex-situ Raman spectra were performed through a confocal Raman microscope (Protrustech Co., Ltd.) using thermoelectrically cooled charge-coupled (CCD) and 50x objective with a laser source of 60 mW in an excitation wavelength of 532 nm. FT-IR spectrophotometry (FTS_3500, Bio-Rad, and USA) was employed to obtain functional group information. Using an SEM (JSM-6500F, JEOL), the morphology and elemental examination of the electrocatalysts were investigated. Without an additional conductive sputter coating, the samples were attached to sticky tape and subject to analysis. The transmission electron microscope TEM (FEI, Tecnai G2-F20) equipped with energy dispersive X-ray spectroscopy (EDX) was used. The copper grids with carbon support films were used to prepare electrocatalyst samples.

The surface chemical state and composition of samples were analyzed by X-ray photoelectron spectroscopy (XPS) ((PHI-1600S) beamline stations located at the Taiwan light source (TLS) of the National Synchrotron Radiation Research Center (NSRRC) in Hsinchu, Taiwan. The incident photon energy was used to record the spectra of Ni 2p, Fe 2p, Ti 2p, O 1s, and S 2p. The pure Au $4f_{7/2}$ core energy at 84.00 eV corresponding to the Fermi energy was used as a reference. The XPS spectra peaks of all elements were fitted using Gaussian XPSPEAK41-software.

The electronic structure and local environments for the Ni, Fe, and Ti K-edge were investigated using an X-ray Absorption Spectroscopy (XAS) beamline station (BL-17C1) located at Taiwan light source, NSRRC in Hsinchu, Taiwan. The operation current of the storage ring of the

electronic accelerator at 360 mA can supply an electronic energy of 1.5 GeV. The measurements were performed in transmission mode at room temperature, with gas-filled ion chambers used as X-ray intensity detectors. A nonlinear least-squares algorithm was applied to the curve fitting of the EXAFS for Ni and Fe K-edge in the r-space. The computer programs were implemented in the Athena 0.8.056 package with the backscattering amplitude. From these analyses, structural parameters, such as coordination number (N), bond distance (R), the Debye-Waller factor ($\Delta\sigma^2$), and the inner potential shift (ΔE_0), were calculated.

Electrochemical Measurements

All electrochemical measurements were performed using a computer-controlled potentiostat (PGSTAT302N, Metrohm Autolab) workstation with a three-electrode system in a 1 M KOH solution that had been purged with argon for 30 min. The working electrode was prepared by applying the as-synthesized electrocatalyst on a Ti foam substrate with dimensions of 1 cm^2 x 1.60 mm (thickness) and a mass loading of 4.3 mg cm^{-2}. The reversible hydrogen electrode (RHE) was the reference electrode, and the graphite rod was the counter electrode. First, the molar ratios of precursors for Ni_3S_2 (Ni: S) and Ni_3Fe_1 LDH (Ni: Fe) growth were optimized and adjusted sequentially, taking into account the electrochemical performance as shown Figure S5g-h. The as-synthesized electrocatalyst were activated by cyclic voltammetry (CV) scans for 100 cycles swept to a potential range between 1 and 1.8 V vs RHE prior to performing any electrochemical testing. The linear sweep voltammetry (LSV) scan rate was recorded at a slow sweep rate of 1 mV s^{-1}. From the LSV data, corresponding Tafel curves were obtained, and the formula $\eta = E_{RHE} - 1.23$ V was used to determine the overpotential for OER. A frequency range of 100 kHz to 0.01 Hz and an AC potential of 5 mV amplitude were used for the evaluation electrochemical impedance spectroscopy (EIS) at 1.5 V. The electrochemical in situ-Raman measurements were performed with a potentiostat (PGSTAT302N, Metrohm Autolab), using a homemade three-electrode system Ag/AgCl (sat. KCl, sat. AgCl), graphite rod, and modified glassy carbon electrode (GCE) were used as a reference, counter electrode, and working electrode, respectively, in the aqueous solution of 1M KOH electrolyte. Chronoamperometry method was used to perform the measurements. All potentials for these measurements are reported versus RHE. The electrochemical stability of the electrocatalyst were evaluated by performing chronopotentiometry measurement under a constant current density of 100 mA cm^{-2}.

Evaluation of Faradaic Efficiency

The Faradaic efficiency (FE) was evaluated in 1.0 M KOH electrolyte using H-type cell to explore the utilization efficiency of electrons involved in the electrochemical process. The FE was calculated using a chronoamperometry stability test to Ni_3Fe_1 LDH/Ni_3S_2/TW for 1 hour at a constant current density of 100 mA cm^{-2}, by comparing experimentally generated oxygen volume with the theoretical oxygen volume by water displacement methods. As shown in Figure S5f, the mol of O_2 gas produced agrees with the theoretical values, and the calculated FE is about 96.1 %. Thus verifying its excellent oxygen conversion efficiency in the OER process. The theoretically generated mole of oxygen (nO_2') was determined using Faraday's laws of electrolysis: $nO_2' = \frac{Q}{4F}$ and $FE = \frac{\textit{actual mole of generated oxygen } (nO2)}{\textit{Theorethically generated mole of oxygen } (nO2')}$, where nO_2 is the actual mole of generated oxygen, Q = It, is measured charge, I is constant current, it is time, and *F* is Faraday constant 96 485 C mol^{-1}. The generated gas products were collected in graduated cylinders filled with water, and the change in the water level in each cylinder indicates the amount of the corresponding gas product generated within a certain amount of time. The water levels were recorded every ten minutes to track the generation of O_2.

Electrochemical Active Surface Area Evaluation

To determine the electrochemical surface area (ECSA), the double-layer capacitance (C_{dl}) was evaluated using cyclic voltammetry (CV) tests in the non-faradaic region at various scan rates ranging from 20 to 100 mV s^{-1}. The C_{dl} is obtained by plotting half of the positive and negative current density differences ($j = j_a - j_c/2$) at a certain potential beside the CV scan rates, which is equals to the value of the linear slopes of the plot. Then, ECSA was computed using Equation (S1) blow.[1]

$$A_{ECSA} = \frac{C_{dl} \textit{ of catalyst } (mF\ cm^{-2})}{\text{Cs } mF\ cm^{-2} \textit{ per } cm^2{}_{ECSA}} \quad \text{(S1)}$$

Where C_s is the specific capacitance of the flat surface (40 µF cm^{-2}) in 1 M alkaline solution.

Catalytic Site Activity

The site activity (SA) refers to the specific catalytic active site on the exposed surface of catalyst at a given potential, which is evaluated as the current per real surface area of the electrocatalyst.[2], which is evaluated according to the previous report.[3]

$\boldsymbol{SA} = \frac{j_\eta}{\mathbf{ECSA}}$, where j_η is the current density (mA) at the constant overpotential (η) (S2)

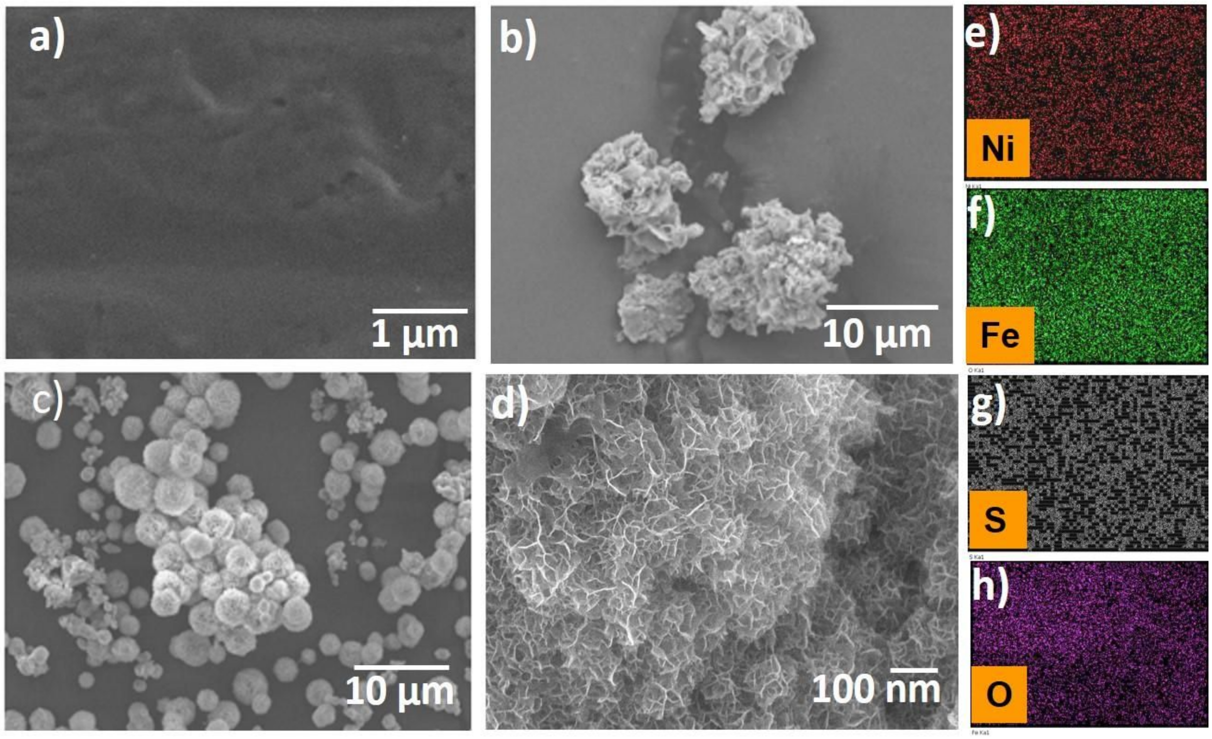

Figure S1. SEM morphology of (a) bare untreated Ti foam (b) Ni_3S_2/TF and (c) Ni_3Fe_1 LDH/TF on the surface of unmodified Ti foam. (d) SEM morphology of Ni_3Fe_1 LDH/Ni_3S_2/TW after stability test and (e-h) SEM elemental mapping images for Ni_3Fe_1 LDH/Ni_3S_2/TW.

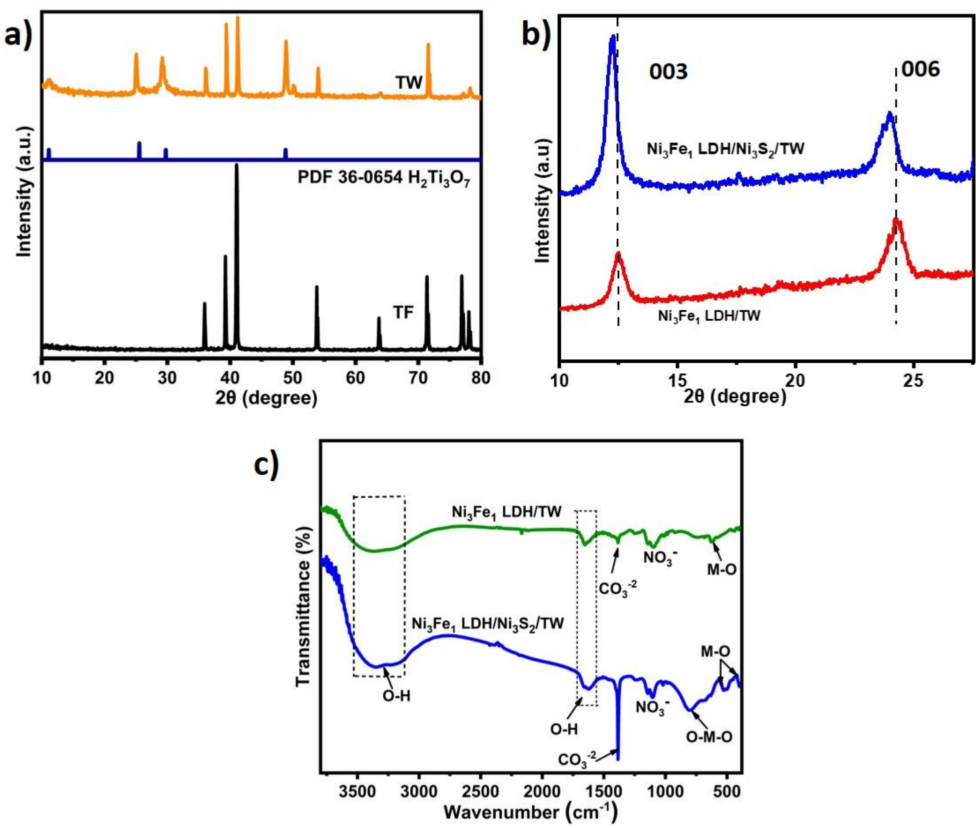

Figure S2. (a) XRD pattern for surface unmodified Ti foam (black) and titanate layers on surface modified titanium foam as Ti web (yellow). (b) Enlarged scale of XRD patterns of Ni_3Fe_1 LDH/TW and Ni_3Fe_1 LDH/Ni_3S_2/TW heterostructure indicating the peak shifts of 003 and 006 planes. **(**c) FT-IR spectra of Ni_3Fe_1 LDH/TW and Ni_3Fe_1 LDH/Ni_3S_2/TW.

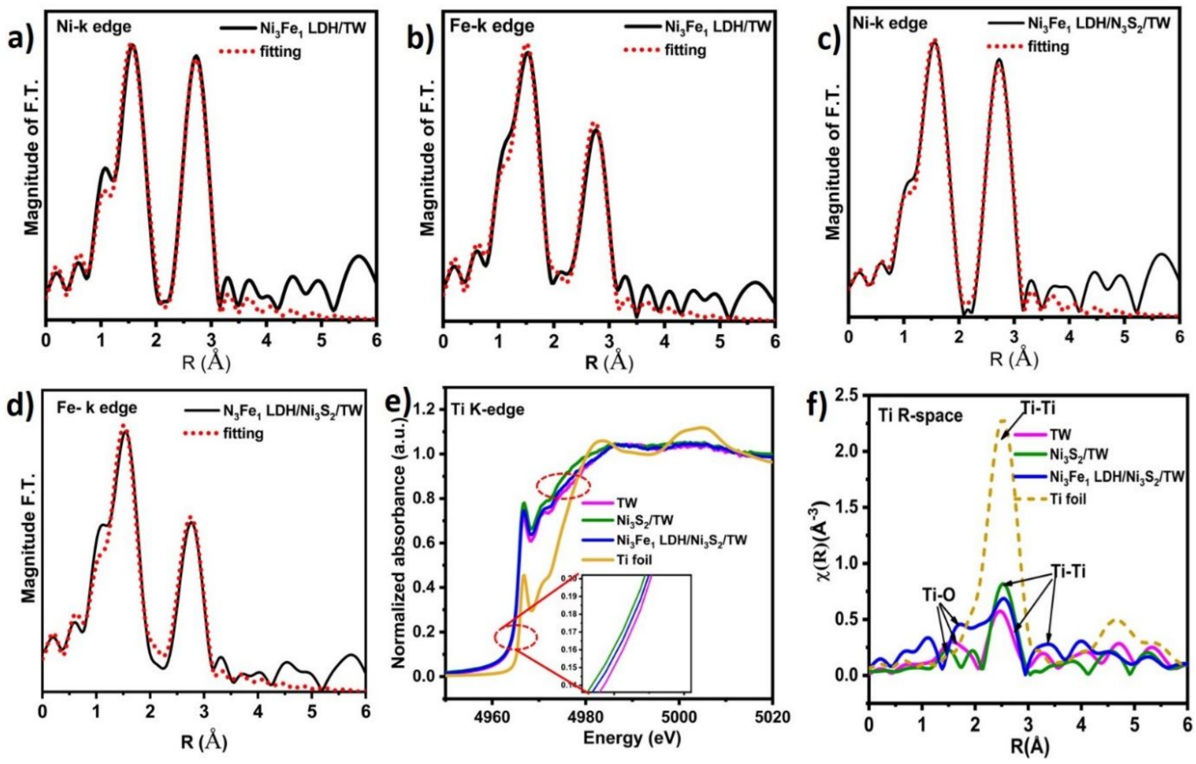

Figure S3. XANES spectra of Ti K-edge (a) and EXAFS spectra of the Ti R-space (b) with corresponding catalysts. Ni and Fe K-edge FT-EXAFS in Ni_3Fe_1 LDH/TW (c, d) and Ni_3Fe_1 LDH/Ni_3S_2/TW (e, f) heterostructure with their respective curve fitting results.

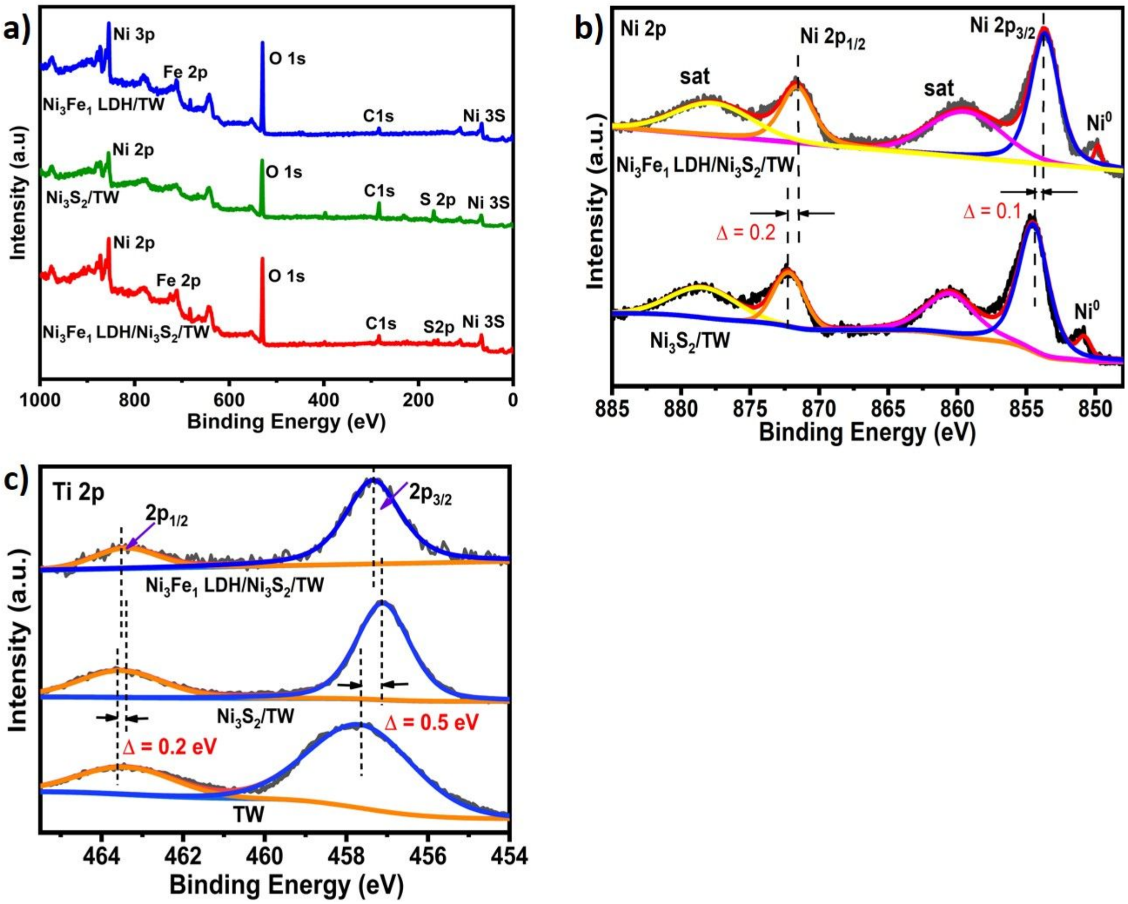

Figure S4. (a) The XPS survey spectra for the corresponding samples. XPS spectra binding energy comparison for (b) Ni 2p of Ni_3S_2/TW and Ni_3Fe_1 LDH/Ni_3S_2/TW. (c) XPS spectra of Ti 2p of as-synthesized electrocatalysts

In Figure S5a-d, CV measurements were performed in the non-faradic potential range where there is no charge transfer reaction, but only adsorption/desorption, with various scan rates to determine the ECSA of the electrocatalyst. A linear trend was constructed by plotting the current density against the scan rate based on the 1 cm^2 geometric surface area of the electrode. C_{dl} was estimated, where the slope is twice of C_{dl}. Finally, the ECSA of the electrocatalyst is estimated from the C_{dl} according to the equation below [2, 3]. ECSA = C_{dl}/C_S, where C_{dl} is the double-layer capacitance from the CV test; C_S is the estimated specific capacitance of a flat surface (0.04 mF cm^{-2}) in a 1.0 M KOH solution.

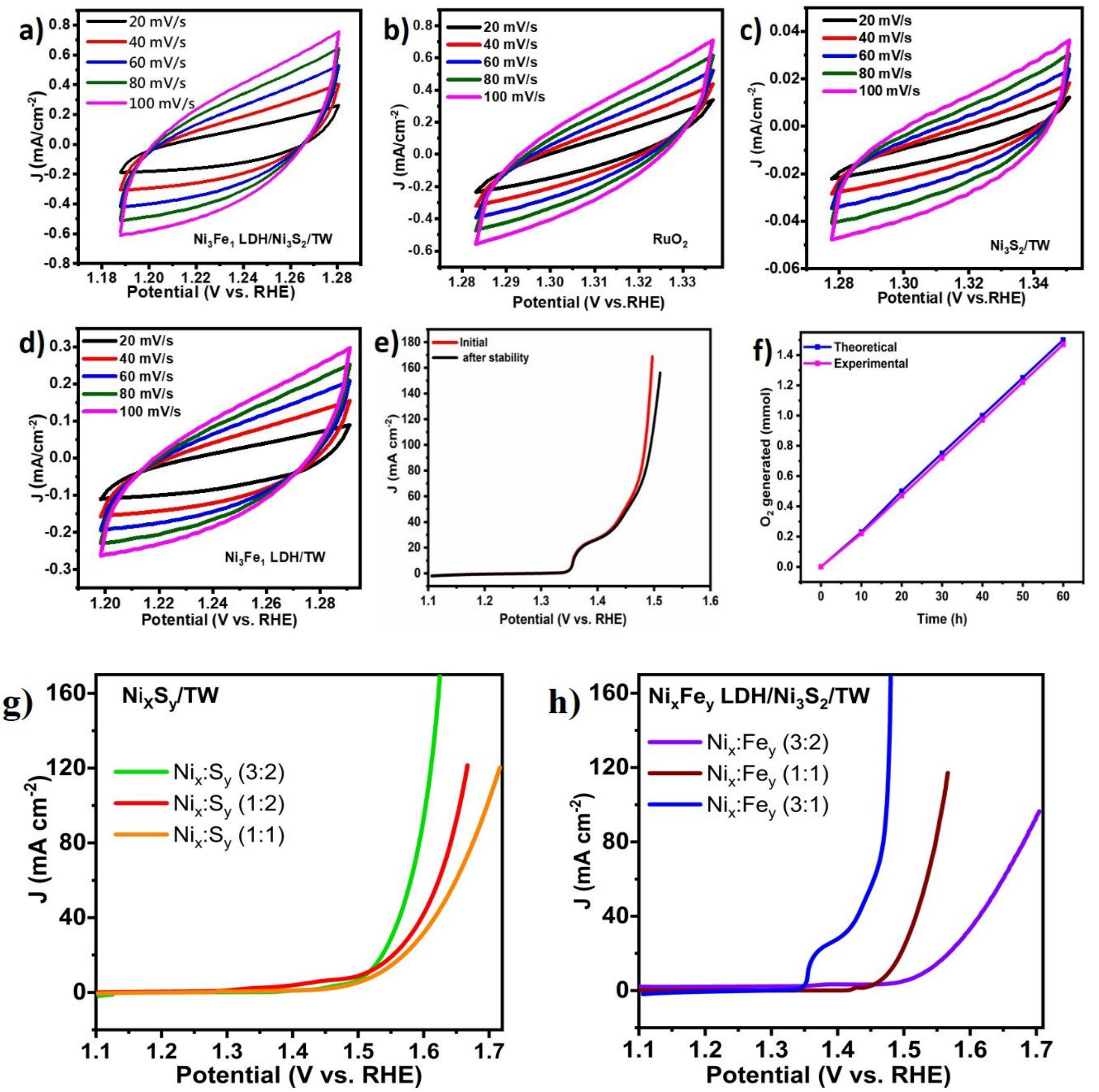

Figure S5. CV for the double-layer capacitance measurements of (a) Ni_3Fe_1 LDH/Ni_3S_2/TW, (b) RuO_2, (c) Ni_3S_2/TW, and (d) Ni_3F_1 LDH/TW electrodes at scan rates of 20-100 mV s^{-1} in 1 M KOH solution. (e) LSV polarization curves of the initial and after stability test for Ni_3Fe_1 LDH/Ni_3S_2/TW in 1 M KOH solution. (f) Experimentally generated oxygen compared with the theoretical amount obtained from the anodic cell by the water displacement method. The OER LSV polarization curves for the molar ratio optimization of (g)Ni_xS_y/TW (Ni_3S_2/TW the best) and (h) Ni_xFe_y LDH/Ni_3S_2/TW (Ni_3Fe_1 LDH/Ni_3S_2/TW the best) electrocatalysts in 1 M KOH at a scan rate of 1 mV s^{-1}

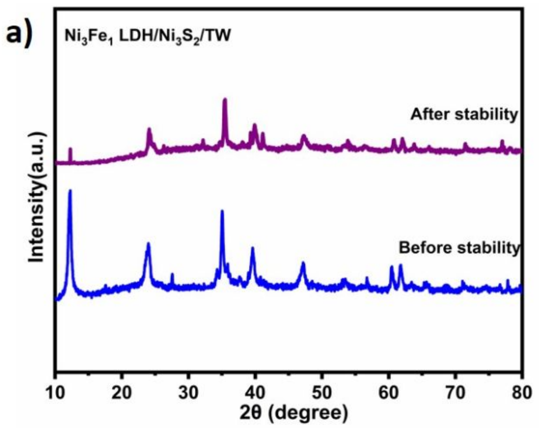

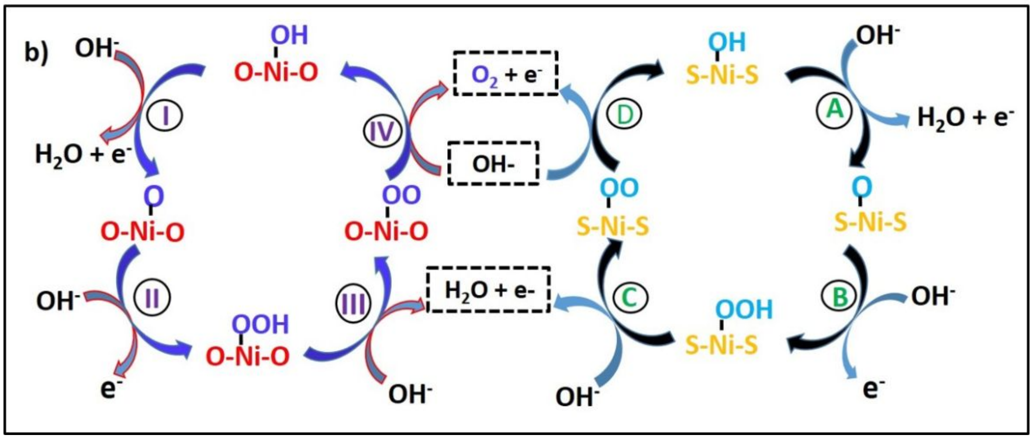

Figure S6. (a) XRD patterns for pre- and post-OER. (b) The proposed OER reaction mechanisms for the synergistic effects of the Ni_3Fe_1 LDH/Ni_3S_2/TW heterostructure

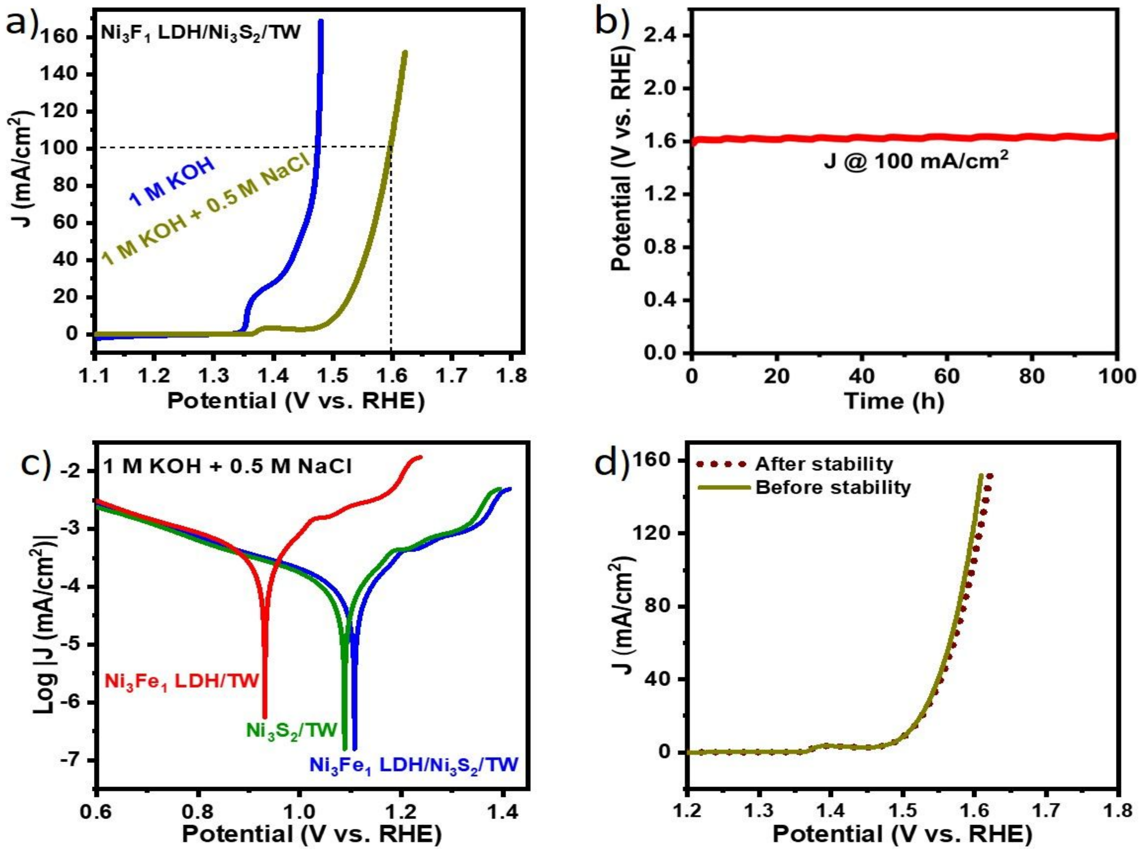

Figure S7. (a) The OER polarization curves of Ni_3Fe_1 LDH/Ni_3S_2/TW electrocatalyst in 1 M KOH and simulated seawater. (b) Chronopotentiometric stability test of the electrocatalyst in simulated seawater at 100 mA cm^{-2}. (c) Linear polarization curves of electrodes comparison for corrosion resistance in simulated seawater as corrosion potential and corrosion current. (d) LSV polarization curves for pre- and post-OER chronopotentiometric stability tests.

Table S1. Local Structure Parameters from EXAFS Fitting results for Ni and Fe K-edge in $Ni(OH)_2$, Ni_3Fe_1 LDH, and Ni_3Fe_1 LDH/Ni_3S_2/TW.

Sample	Shell	N	R (Å)	ΔE_0 (eV)	σ^2 (Å^2)	R-factor (x10^{-3})
$Ni(OH)_2$	Ni-O	6.0±0.6	2.04±0.01	-8.6	0.006	1.1
	Ni-Ni	6.0±0.9	3.10±0.01	-4.3	0.008	
Ni_3Fe_1 LDH/TW	Ni-O	5.5±0.7	2.04±0.01	-6.4	0.005	2.8
	Ni-Ni	3.6±0.6	3.10±0.02	-4.3	0.007	
	Ni-Fe	1.8±0.3	3.08±0.03	-4.3	0.007	
	Fe-O	6.0±1.4	1.98±0.02	-8.7	0.007	
	Fe-Ni	5.4±0.9	3.08±0.03	-3.9	0.009	
Ni_3Fe_1 LDH/Ni_3S_2/TW	Ni-O	4.7±0.6	2.03±0.01	-7.3	0.005	4.1
	Ni-Ni	3.0±0.5	3.10±0.02	-3.7	0.007	
	Ni-Fe	1.5±0.3	3.08±0.02	-3.7	0.007	
	Fe-O	5.2±0.9	1.99±0.02	-5.7	0.007	
	Fe-Ni	4.5±0.9	3.08±0.02	-2.4	0.009	

N = coordination number; R = distance between absorber and backscatter atoms; σ^2 = Debye-Waller factor; ΔE_0 = energy shift

The d-spacing and crystallite sizes (Table S2) were obtained from the full width of half maximum (FWHM) of XRD data using the Scherer equation of the reflections indexed as (003), (006).

Table S2. Crystalline size, ECSA, site activity and basal spacing were estimated from (003) and (006) diffraction patterns for the XRD obtained for Ni_3Fe_1 LDH/TW, Ni_3Fe_1 LDH/Ni_3S_2/TW electrocatalysts.

**Electrocatalysts**	**Crystallite size (nm)**	**d-spacing (nm)**				**ECSA (cm^2)**	**Site activity (μA)**
		**2θ**	**003**	**2θ**	**006**		
Ni_3Fe_1 LDH/TW	15.86	12.4	0.704	24.2	0.342	31.5	126
Ni_3Fe_1 LDH/Ni_3S_2/TW	14.43	12.1	0.719	23.8	0.361	89.75	557
Ni_3S_2/TW						6.75	397

Table S3 compares the activity of Ni_3Fe_1 LDH/Ni_3S_2/TW heterostructure with previous reports in the literature. Ni_3Fe_1 LDH/Ni_3S_2/TW electrocatalyst for OER show the lowest Tafel slope, reasonable overpotential, and the most stable performances.

Table S3. Summary of OER activities of relevant electrocatalysts compared to recently reported literature

**Electrocatalysts**	**Subst rate**	**J (mA cm^{-2})**	**Over potential (mV)**	**Tafel slope (mV dec^{-1})**	**Electrolyte (1.0 M)**	**Stability (hr)**	**Ref.**
Ni_3Fe_1 LDH/Ni_3S_2/TW	TF	100	220	42.3	KOH	100	This study
CeO_{2-x}@CoFe LDH/NF	NF	100	224	23.7	KOH	30	[4]
S-NiCoFe LDH/CC	CC	100	258	46	KOH	30	[5]
Ni_3S_2@NiCo-LDH	NF	100	346	47.5	KOH	24	[6]
NiTe/NiCo-LDH/NF	NF	100	376	76.5	KOH	24	[7]
Ni_3S_2/NiFe LDH	NF	100	233	29.71	KOH	40	[8]
NiFe-LDH/Ni_9S_8/NF	NF	100	265	38.1	KOH	60	[9]
NiCo LDH/NiCoS/CC	CC	100	308	48	KOH	25	[10]
$MnCo_2O_4$@NiFe-LDH/NF)	NF	100	283	84.1	KOH	20	[11]
NiTe/NiS	NF	100	257	49	KOH	50	[12]
NiFeV LDHs/NF	NF	100	233	42	KOH	18	[13]
NiFe-Cu-LDHs.	CC	100	250	-	KOH	25	[14]

Table S4 Pre- and post-OER XPS O 1s spectra peak area ratios for Ni_3Fe_1 LDH/Ni_3S_2/TW electrocatalyst.

Oxygen intermediate species	Peak Area (a. u.)		Peak area ratio (%)	
	Pre-OER	Post-OER	Pre-OER	Post-OER
**H_2O**	17433	26143	39.8%	39.3%
**M-OH**	17796	20376	40.6%	30.6%
**M-O**	8595	20020	19.6%	30.1%

References

1. Yang, R.; Zhou, Y.; Xing, Y.; Li, D.; Jiang, D.; Chen, M.; Shi, W.; Yuan, S., Synergistic Coupling of CoFe-LDH Arrays with NiFe-LDH Nanosheet for Highly Efficient Overall Water Splitting in Alkaline Media. *Appl. Catal. B: Environ.* **2019,** *253*, 131-139.

2. Wei, C.; Sun, S.; Mandler, D.; Wang, X.; Qiao, S. Z.; Xu, Z. J., Approaches for Measuring the Surface Areas of Metal Oxide Electrocatalysts for Determining their Intrinsic Electrocatalytic Activity. *Chem. Soc. Rev.* **2019,** *48* (9), 2518-2534.

3. Liu, Y.; Bai, Y.; Yang, W.; Ma, J.; Sun, K., Self-Supported Electrode of NiCo-LDH/$NiCo_2S_4$/CC with Enhanced Performance for Oxygen Evolution Reaction and Hydrogen Evolution Eeaction. *Electrochim. Acta* **2021,** *367*, 137534.

4. Hu, Y.; Liu, W.; Jiang, K.; Xu, L.; Guan, M.; Bao, J.; Ji, H.; Li, H., Constructing a CeO_{2-x}@CoFe-Layered Double Hydroxide Heterostructure as an Improved Electrocatalyst for Highly Efficient Water Oxidation. *Inorg. Chem. Front.* **2020,** *7* (22), 4461-4468.

5. Cao, L.-M.; Wang, J.-W.; Zhong, D.-C.; Lu, T.-B., Template-Directed Synthesis of Sulphur Doped NiCoFe Layered Double Hydroxide Porous Nanosheets with Enhanced Electrocatalytic Activity for the Oxygen Evolution Reaction. *J. Mater. Chem. A* **2018,** *6* (7), 3224-3230.

6. Kitiphatpiboon, N.; Chen, M.; Li, X.; Liu, C.; Li, S.; Wang, J.; Peng, S.; Abudula, A.; Guan, G., Heterointerface Engineering of Ni_3S_2@NiCo-LDH Core-Shell Structure for Efficient Oxygen Evolution Reaction Under Intermittent Conditions. *Electrochim. Acta* **2022,** *435*, 141438.

7. He, B.; Zhao, P.; Pan, G.-X.; Lu, Q.; Li, H.-Q.; Ye, F.; Tang, Y.-W.; Hao, Q.-L.; Su, Z., Interface Engineering of NiTe/NiCo-LDH Core-Shell Structure to Enhance Oxygen Evolution Electrocatalysis Performance. *J. Alloys Compd.* **2023,** *938*, 168673.

8. Liang, Z.; Zhou, P.; Wang, Z.; Wang, P.; Liu, Y.; Qin, X.; Zhang, X.; Dai, Y.; Zheng, Z.; Huang, B., Electrodeposition of NiFe Layered Double Hydroxide on Ni_3S_2 Nanosheets for Efficient Electrocatalytic Water Oxidation. *Int. J. Hydrog. Energy* **2020,** *45* (15), 8659-8666.

9. Yao, Y.; Hu, E.; Zheng, H.; Chen, Y.; Wang, Z.; Cui, Y.; Qian, G., Scalable Synthesis of NiFe-LDH/Ni_9S_8/NF Nanosheets by Two-Step Corrosion for Efficient Oxygen Electrocatalysis. *ChemCatChem* **2022,** *14* (1), e202101280.

10. Li, J.; Wang, L.; He, H.; Chen, Y.; Gao, Z.; Ma, N.; Wang, B.; Zheng, L.; Li, R.; Wei, Y., Interface Construction of NiCo LDH/NiCoS Based on the 2D Ultrathin Nanosheet Towards Oxygen Evolution Reaction. *Nano Res.* **2022,** *15* (6), 4986-4995.

11. Shi, H.; Yang, K.; Wang, F.; Ni, Y.; Zhai, M., Hierarchical $MnCo_2O_4$ Nanowire@NiFe Layered Double Hydroxide Nanosheet Heterostructures on Ni Foam for Overall Water Splitting. *CrystEngComm* **2021,** *23* (40), 7141-7150.

12. Xue, Z.; Li, X.; Liu, Q.; Cai, M.; Liu, K.; Liu, M.; Ke, Z.; Liu, X.; Li, G., Interfacial Electronic Structure Modulation of NiTe Nanoarrays with NiS Nanodots Facilitates Electrocatalytic Oxygen Evolution. *Adv. Mater.* **2019,** *31* (21), 1900430.

13. Li, P.; Duan, X.; Kuang, Y.; Li, Y.; Zhang, G.; Liu, W.; Sun, X., Tuning Electronic Structure of NiFe Layered Double Hydroxides with Vanadium Doping Toward High Efficient Electrocatalytic Water Oxidation. *Adv. Energy Mater.* **2018,** *8* (15), 1703341.

14. Enhtuwshin, E.; Mhin, S.; Kim, K. M.; Ryu, J. H.; Kim, S. J.; Jung, S. Y.; Kang, S.; Choi, S.; Han, H., Ni-Fe-Cu-layered Double Hydroxides as High-Performance Electrocatalysts for Alkaline Water Oxidation. *Int. J. Energy Res.* **2021,** *45* (10), 15312-15322.
